# The horizontal transfer of *Pseudomonas aeruginosa* PA14 ICE PAPI-1 is controlled by a transcriptional triad between TprA, NdpA2 and MvaT

**DOI:** 10.1093/nar/gkab827

**Published:** 2021-10-13

**Authors:** Gauthier Dangla-Pélissier, Nicolas Roux, Victoria Schmidt, Gaël Chambonnier, Moly Ba, Corinne Sebban-Kreuzer, Sophie de Bentzmann, Caroline Giraud, Christophe Bordi

**Affiliations:** LISM, IMM, Aix-Marseille University, Marseille 13402, France; LISM, IMM, Aix-Marseille University, Marseille 13402, France; LISM, IMM, Aix-Marseille University, Marseille 13402, France; LISM, IMM, Aix-Marseille University, Marseille 13402, France; LISM, IMM, Aix-Marseille University, Marseille 13402, France; LISM, IMM, Aix-Marseille University, Marseille 13402, France; LISM, IMM, Aix-Marseille University, Marseille 13402, France; U2RM Stress/Virulence, Normandy University, UNICAEN, 14000 Caen, France; LISM, IMM, Aix-Marseille University, Marseille 13402, France

## Abstract

*Pseudomonas aeruginosa* is a major cause of nosocomial infections, particularly in immunocompromised patients or in individuals with cystic fibrosis. Genome sequences reveal that most *P. aeruginosa* strains contain a significant number of accessory genes gathered in genomic islands. Those genes are essential for *P. aeruginosa* to invade new ecological niches with high levels of antibiotic usage, like hospitals, or to survive during host infection by providing pathogenicity determinants. *P. aeruginosa* pathogenicity island 1 (PAPI-1), one of the largest genomic islands, encodes several putative virulence factors, including toxins, biofilm genes and antibiotic-resistance traits. The integrative and conjugative element (ICE) PAPI-1 is horizontally transferable by conjugation via a specialized GI-T4SS, but the mechanism regulating this transfer is currently unknown. Here, we show that this GI-T4SS conjugative machinery is directly induced by TprA, a regulator encoded within PAPI-1. Our data indicate that the nucleotide associated protein NdpA2 acts in synergy with TprA, removing a repressive mechanism exerted by MvaT. In addition, using a transcriptomic approach, we unravelled the regulon controlled by Ndpa2/TprA and showed that they act as major regulators on the genes belonging to PAPI-1. Moreover, TprA and NdpA2 trigger an atypical biofilm structure and enhance ICE PAPI-1 transfer.

## INTRODUCTION

Beyond the core genome of human pathogenic bacteria, increasing data emanating from genome sequence projects illustrate that closely related strains contain many additional accessory genes that have been acquired through horizontal gene transfer (HGT), leading to evolution through diversification and adaptation of microorganisms. These accessory genes can form syntenic blocks with specific gene functions, such as metabolism, symbiosis, fitness, resistance or pathogenicity, and can be identified by the presence of signature features (1). Among them is an atypical nucleotide composition relative to the rest of the genome, a location within predicted sites of chromosomal integration (att sites), and the presence of genes encoding conjugation machineries or bacteriophages. The acquisition by HGT of this accessory genome could dramatically change the threat of a microorganism (2,3). For example, acquisition of the OI-57 genomic island by *Escherichia coli* species allows the microorganism to cause haemorrhagic colitis, leading to the development of the potentially fatal complication haemolytic uremic syndrome (2).

**Figure 1.**
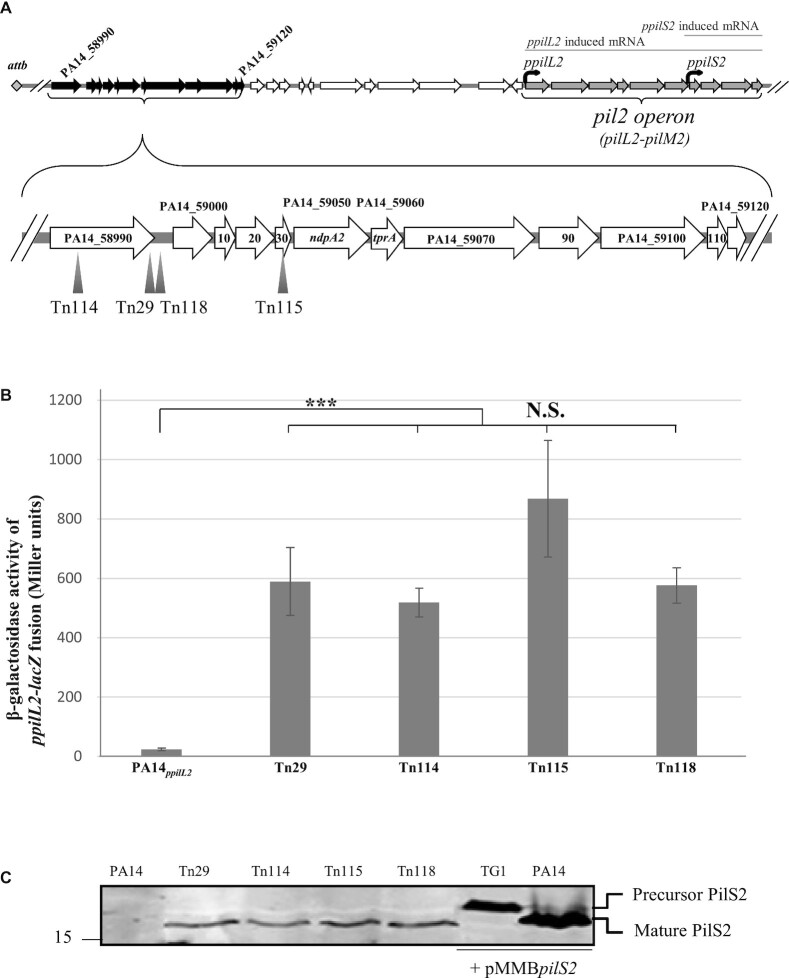
Identification of the PA14_58990-PA14_59120 locus as a regulator of *pil2* operon expression. (**A**) Localization of mariner transposon insertion inside the PA14_58990-PA14_59120 locus. (**B**) Expression of the *ppilL2–lacZ* chromosomal fusion was monitored in the PA14, Tn29, Tn114, Tn115 and Tn118 strains after 7 h of growth in LB. Corresponding β-galactosidase activities are expressed in Miller units and correspond to mean values (with error bars) obtained from three independent experiments. Wilcoxon–Mann–Whitney tests were performed, and N.S. and *** indicate to non-significant, and *P* < 0.001, respectively. (**C**) Detection of PilS2 pilin production in whole-cell extracts from PA14, Tn29, Tn114, Tn115 and T118 strains. TG1 *E. coli* and *P. aeruginosa* PA14 strains bearing the pMMB*pilS2* plasmid permitting the overexpression of the *pilS2* gene were used, respectively, as a positive control of a precursor and mature form of PilS2 protein. The number on the left indicates the sizes of the molecular mass standards (in kDa). Functions of the genes are listed in Supplementary Table S4.

*Pseudomonas aeruginosa*, a Gram-negative environmental bacterium responsible for opportunistic infections, has become a major cause of nosocomial infections worldwide (about 10% of all such infections in most European Union hospitals) and a serious threat to public health (4). Previous studies comparing *Pseudomonas aeruginosa* genomes showed that HGT plays an important role in its evolution (5). Sequencing several strains (6–9) showed that in addition to a conserved core genome, variable accessory genes are also found that are largely associated with genomic islands. The PA14 strain, a highly virulent human isolate capable of infecting multiple hosts, possesses two pathogenicity islands (PAPI-1 and PAPI-2), which are absent from the PAO1 reference genome. PAPI-1 and PAPI-2 contribute, individually and synergistically, to the increased virulence of the PA14 strain in acute pneumonia and bacteraemia animal models (10). The 108 kb pathogenicity island PAPI-1 is the larger island and belongs to the integrative and conjugative element (ICE) family. ICEs are mobile genetic elements classically found integrated in the host genome that can be passively propagated during chromosomal replication or horizontally transferred to a recipient (11). PAPI-1 contains 115 genes and is integrated into the host chromosome at the *attB* site juxtaposed to *tRNA-lys* genes. This ICE is a mosaic of modular genetic elements, containing several functional modules called cargo modules. Each of these cargos embed genes involved in the same function; among them are pathogenicity-related genes, including: the PA14_59710–59760 gene cluster, which is important for mouse infection; a gene cluster related to biofilm synthesis (CupD fimbriae); and the sRNA PesA involved in post-transcriptional regulation of Pyocin S3 or the PvrS/PvrR, which is a two-component system involved in biofilm synthesis and antibiotic resistance (10,12–14). Most of the remaining PAPI-1 genes encode functions related to DNA mobilization, conjugation, integration and partition activities (7). Thanks to these genes, PAPI-1 can be mobilized from a PA14 donor strain to any recipient *P. aeruginosa* strain lacking it, using conjugative machinery belonging to the GI type IV secretion system (GI-T4SS) family (15–19). At the genetic level, the conjugative pilus is encoded by an operon called the *pil2* operon, which contains 10 genes (*pilL2-pilM2*) and presents two putative promoters localized upstream of the *pilL2* and *pilS2* genes (15) (Figure 1A). Nevertheless, ICE PAPI-1 cannot be considered a truly self-transmissible element. Indeed, PilS2, the major subunit of the pilus, is synthesized as a precursor with a 14 amino acid leader peptide and needs the action of PilD prepilin peptidase, encoded by the chromosome host, to be matured (15). This PilS2 maturation dependency on a host-encoded prepilin peptidase restricts PAPI-1 transmission only to donors that express a compatible prepilin peptidase. PAPI-1 can be excised from its tRNA att site and a copy can be transferred and integrated into the host chromosome at any of the two tRNA-Lys of a recipient strain. A remarkable feature of PAPI-1 transmission is the existence of an exclusion mechanism that restricts the ICE transfer to recipient cells devoid of PAPI-1. This exclusion mechanism is based on the recognition of common polysaccharide antigen (CPA) lipopolysaccharides of recipient cells by the conjugative pilus to initiate the ICE PAPI-1 transfer. Once integrated, and to exclude acquisition of any additional copies of PAPI-1, the receiver strain produces less CPA, blocking the possibility of being recognized by the GI-T4SS pilus a second time (19).

Expression of excision/integration and conjugation genes is usually tightly regulated to maintain ICE genes as quiescent as possible in the host (20). Indeed, constitutive expression of these genes has been observed to be detrimental, especially for the host and the maintenance of the ICE. For example, a constitutive activation of ICE*clc* from *Pseudomonas* sp. strain B13 has been shown to cause a growth reduction, leading to the death of the cell host (21). More generally, it has been shown that, in some Gram-negative bacteria, constitutive expression of a mating pilus may increase host susceptibility to phage infection (22). These deleterious effects lead to a selective pressure against ICE gene activation to leave them as quiescent as possible. However, to permit their transfer, ICE-encoded genes need to be induced, and a large variety of signals has been described to be involved in this process. These signals are not universally conserved, but there is some common theme, for example, the SOS response, cell–cell signalling, the stationary phase or antibiotic pressures (23). ICEs are self-transmissible and, generally, the regulators controlling their transfer are encoded in the ICEs themselves (24,25). For example, the transfer regulation of ICE*MlsymR7A*, a large ICE usually found in rhizobia, is controlled by a quorum sensing mechanism encoded on it (25). In some cases, host-encoded elements also participate in the ICE transfer, such as in the case of ICEs SXT-R391 and ICE*Bs1*, whose transfer is induced by the SOS response through RecA-dependent activation (24,26,27). Our knowledge about PAPI-1 transfer regulation is still limited regarding the environmental cues and the regulatory elements involved in this process. Carter and collaborators observed that in the stationary growth phase, the expression of the two *pil2* operon promoters is increased, suggesting that PAPI-1 transfer could be regulated by a stationary-phase regulator. A sequence analysis of the two predicted promoters shows a putative Crp binding site in the first putative promoter of the *pilL2* gene, suggesting that the expression of the GI-T4SS machinery, and therefore the conjugation, may be subject to regulation by cyclic AMP (cAMP). Moreover, the regulation of these two promoters has been suggested to not require any of the PAPI-1-encoded regulators, because the expression of the promoter follows the same regulatory profile in PA14 and PAO1, which is devoid of PAPI-1 (15).

In this work, we demonstrated for the first time that the *P. aeruginosa* PA14 conjugative machinery is directly induced by PA14_59060 (TprA), a PAPI-1-encoded transcription factor. Our data also indicate that the nucleotide associated protein PA14_59050 (NdpA2) acts in synergy with TprA in this regulation process by releasing MvaT repression. Moreover, using a transcriptomic approach, we unravelled the regulon controlled by TprA and we demonstrated that TprA is a major regulator of ICE PAPI-1 genes, triggering PAPI-1 transfer and biofilm formation.

## MATERIALS AND METHODS

### Bacterial strains and growth conditions

The strains and plasmids used in this study are listed in Supplementary Tables S1 and S2, respectively. Strains were grown at 37°C in lysogeny broth (LB) medium. Recombinant plasmids were introduced into *P. aeruginosa* through conjugative transfer, using pRK2013 (28). Transconjugants were selected on *Pseudomonas* isolation agar (PIA, Difco Laboratories) supplemented with appropriate antibiotics. The following antibiotic concentrations were used: (i) for *Escherichia coli*: 100 μg/ml ampicillin (Ap), 25 μg/ml gentamicin (Gm), 25 μg/ml kanamycin (Km), 15 μg/ml tetracycline (Tc) and 50 μg/ml streptomycin (Sm); (ii) for *P. aeruginosa*: 300 μg/ml carbenicillin (Cb), 50 μg/ml Gm, 200 μg/ml Tc and 2 mg/ml Sm.

### Chromosomal transcriptional fusions

The DNA fragment containing the putative promoter region of the *pil2* locus (521 bp) was amplified by polymerase chain reaction (PCR), with the MiniCTX-*ppilL2-lacZ*-1/MiniCTX-*ppilL2-lacZ*-2 oligonucleotide pair (see Supplementary Table S5). The resulting DNA fragment was digested with HindIII and PstI restriction enzymes and inserted into the miniCTX-*lacZ* vector, generating the miniCTX-*ppilL2-lacZ* construct. This plasmid was used to generate a chromosomal *ppilL2-lacZ* fusion in the PA14 strain, as previously described. The *FRT* cassette-excision step was performed as previously described (29), resulting in the generation of the PA14*attB::ppilL2-lacZ* (PA14*_ppilL2_*) strain. A similar procedure was used to generate the PA14*_ppilL2__Δ__tprA-BS_* strain from the miniCTX-*ppilL2_Δ__tprA-BS_-lacZ* plasmid. The latter was generated using the mega-priming method and MiniCTX-*ppilL2_Δ__tprA__-BS_*-*lacZ*-1/MiniCTX-*ppilL2_Δ__tprA__-BS_*-*lacZ*-2 oligonucleotide pairs on the miniCTX-*ppilL2-lacZ* construct, as previously described (30).

### β-Galactosidase activity monitoring

Strains carrying *ppilL2*-*lacZ* and *ppilL2_Δ__tprA-BS_-lacZ* reporter fusions were grown at 37°C in LB medium with shaking. Bacterial cells were collected by centrifugation at different growth times. The β-galactosidase activity was measured using the Miller method (31).

### Gene inactivation by stop codon addition

Because many genes examined in this work belong to operons or overlap other genes, we have adopted a gene inactivation strategy in which we have inserted one or two stop codons in the open reading frame (ORF) of the targeted genes, at the chromosomal level, to avoid polar effects on neighbouring genes. To generate the different chromosomal substitutions, DNA fragments corresponding to the upstream and downstream sequences (∼500 bp) of the target region where we want to add a stop codon were amplified from PA14 genomic DNA using appropriate oligonucleotide pairs (see Supplementary Table S5). The upstream and downstream PCR products were cloned into a BamHI linearized pKNG101 suicide vector using the one-step sequence and ligation-independent cloning (SLIC) strategy (32), yielding pK-*ndpA2_P2Stop_*, pK-*tprA_A4Stop_*, pK-*ihfα_T5__Stop_*, pK-*ihfβ_Q32StopM33Stop_*, pK-*dps_I7StopG8Stop_*, pK-*fis_T8Stop_*, pK-*hupα_A7StopA8Stop_*, pK-*hupβ_I7StopD8Stop_*, pK-*mvaT_Y7StopR8Stop_* and pK-*mvaU_F7StopR8Stop_*. To perform PA14 chromosome editing, the resulting suicide plasmids were introduced into the genomic DNA through conjugative transfer by a three-partner procedure using the *E. coli* pRK2013 strain. To construct the M0Δ*tprA* strain where the *tprA* ORF is interrupted by a stop codon, the suicide plasmid PK-*tprA_A4Stop_* was introduced in the M0 strain by a three-partner mating. Substitution mutants were obtained by a double selection: first on LB agar supplemented with Irgasan (25 μg/ml) and Sm (2000 μg/ml) at 37°C, followed by NaCl-free LB agar containing 6% sucrose at 20°C. Each mutation was checked by sequencing (Eurofins Genomics).

### Plasmid construction

Plasmids pBBR-*ndpA2*, pBBR-*tprA*, pBBR-*ndpA2*-*tprA*, pBBR-*mvaT*, pMMB*pilS2*, pLIC-*ndpA2*, pLIC-*mvaT* and pLIC-*tprA* were constructed as follows. Briefly, DNA fragments encompassing each ORF were amplified by PCR using the PA14 chromosome as a matrix with the appropriate oligonucleotide pairs (see Supplementary Table S5). After purification, the PCR products were inserted into their destination vector using the SLIC strategy (32).

### Transposon mutagenesis

The mini transposon vector pBT20, which was used for mutagenesis, contains a p*tac* promoter that, according to its orientation, can drive expression of downstream genes. The transposition of the mini transposon is catalysed by the Himar-1 mariner transposase. We spotted 50 μl of overnight cultures of the donor strain containing pBT20 (*E. coli* Sm10) and the helper strain (*E. coli* 1047/pRK2013), in triplicate, on dry LB agar plates, which were then incubated at 37°C for 2 h, while the recipient PA14*_ppilL2_* was incubated at 42°C. We added 100 μl of the recipient strain to each spot and the plate was incubated at 37°C for 4 h. The duration of tripartite mating was optimized to maximize the number of trans-conjugants but to minimize the number of daughter cells. The spots were scraped off and re-suspended in 1 ml of LB medium. The resulting suspension was plated on LB agar plates (300 μl on a 135 mm plate) containing 25 μg/ml Irgasan, 80 μg/ml X-gal, 20 μM FeSO_4_ and 75 μg/l Gm. We obtained 1.5 × 10^5^ colonies from 81 plates and screened for blue colonies.

### Transposon localization

The transposon insertion site was determined systematically for the mutants, which gave blue colonies; the wild-type parental clone remaining white. A semi-random PCR method was used, involving cell lysis at 95°C, initial amplification of the sequences adjacent to the transposon insertion with a transposon-specific primer and an arbitrary primer (pBT20-1 and ARB1D-Aus primers, respectively), followed by a second amplification with a nested transposon-specific primer and a primer corresponding to a non-random portion of the arbitrary primer used in the first PCR (pBT20-2 and ARB2A-Aus primers, respectively) (33). The PCR products were purified with the Macherey-Nagel NucleoSpin^®^ Gel and PCR Clean-up kit and sequenced by Eurofins Genomics using the pBT20-2 transposon-specific primer.

### Construction of strains with mariner transposon insertion

To generate the strains M0 to M3 into which we inserted part of the mariner transposon at different locations within the PA14_59030-PA14_59120 locus of strain PA14*_ppilL2_*, we proceeded as follows. First, the DNA fragments corresponding to the upstream and downstream sequences (∼500 bp) of the insertion region were amplified from the PA14 genomic DNA using the appropriate oligonucleotide pairs (see Supplementary Table S5). Next, the mariner fragment was amplified from Tn115 genomic DNA using the M-1/M-2 oligonucleotide pair. Finally, the upstream, downstream and mariner PCR products were cloned into a BamHI linearized pKNG101 suicide vector using the SLIC strategy, yielding pK-M0, pK-M1, pK-M2 and pK-M3 pKNG101-based plasmids. To insert the transposon in the chromosome, the resulting suicide plasmids were introduced into the PA14*_ppilL2_* strain by a three-partner mating using pRK2013 *E. coli* strain. Transposon insertion was achieved by a double selection: first on LB agar supplemented with Irgasan (25 μg/ml) and Sm (2000 μg/ml) at 37°C, followed by NaCl-free LB agar containing 6% sucrose at 20°C. Each mutation was checked by sequencing (Eurofins Genomics).

### Quantitative real time polymerase chain reaction (qRT-PCR)

Three independent cultures of PA14 strains bearing pBBR1MCS4, pBBR-*ndpA2*, pBBR-*tprA* or pBBR-*ndpA2*-*tprA* plasmids were grown under agitation at 37°C and harvested after 7 h of growth. Total cellular RNA from a 5 ml culture was isolated using the PureYield RNA Midiprep System (Promega), cleaned up and concentrated using the RNeasy Micro Kit (Qiagen). The yield, purity and integrity of RNA were further evaluated on Nanodrop (Nanodrop Technologies) and Experion (Bio-Rad) devices. Further, DNA contamination of prepared RNA was assessed using the Access RT-PCR system (Promega) and the oligonucleotide pairs listed in Supplementary Table S5. Reverse transcription was performed on 2 μg of RNA using the SuperScriptIII first strand synthesis system (Invitrogen). The qRT-PCR cycling parameters were: 98°C for 2 min; 45 cycles of 98°C for 5 s and 60°C for 10 s; and 10 min at 95°C. To determine the amplification kinetics of each product, the fluorescence derived from the incorporation of EvaGreen into the double-stranded PCR products was measured at the end of each cycle using SsoFast EvaGreen Supermix (Bio-Rad). The Relative Expression Software Tool (REST) was used to analyse the data (34) and the *16S* gene was used as reference for normalization.

### Transcriptional profiling using microarrays

The experiment comparing M0 and Tn38 transcriptomes (M0/Tn38) and the experiment comparing M0 and M0Δ*tprA* transcriptomes (M0/M0Δ*tprA*) were carried out independently twice for M0/Tn38 and three times for M0/M0ΔtprA. Bacteria were grown in LB under agitation at 37°C and were harvested during the beginning of the stationary phase (7 h, OD_600_ = 3.4) using centrifugation at 4°C. The samples were quickly processed to prepare RNA using the PureYield RNA Midiprep System (Promega), cleaned up and concentrated using the RNeasy Micro Kit. The yield, purity and integrity of RNA were checked by the Experion automated electrophoresis system and the absence of DNA contamination was verified by PCR. RNA samples (500 ng) were further processed for double Cy3/Cy5 labelling using the ChipShot Direct Labelling and Clean-Up System kits (Promega). Tn38 and M0Δ*tprA* samples were labelled with Cy3 and M0 samples were labelled with Cy5. Briefly, 500 ng of RNA was first hybridized with hexameric random primers. Then, dNTPs (330 nM each) were added together with 1 mM of Cy3 or Cy5 (Amersham) and 200 units of ChipShot reverse transcriptase. The mixture was incubated for 2 h at 42°C and the reaction was stopped by a 15 min incubation at 37°C with RNAse. Unincorporated nucleotides were removed by using the ChipShot Direct Labelling and Clean-Up System (Promega). Dye incorporation rates were measured using a Nanodrop® ND-1000 spectrophotometer. Cy3- and Cy-5 labelled cDNA mixtures (300 ng) were hybridized for 17 h at 65°C, according to Agilent protocols, on Multi-genome *P. aeruginosa* (MGPA) DNA Chips.

### Microarray analysis

Data were further treated with R (35) using the LIMMA package from Bioconductor (36,37). Intra-array normalization was performed using the local Lowess (Loess) regression method with ‘Normexp’ background noise correction, whereas inter-array normalization was performed using a quantile method. Differentially expressed genes were extracted using the LIMMA package, using linear regression (lmFit), Bayesian models (eBayes) and Benjamini–Hochberg multiple-test correction (BH). We considered significance in the different replicates for a *P*-value <0.05. Lists of genes were further restricted, applying a log_2_fold-change of 1.584 (∼3-fold) induction or repression criterion. The microarray dataset is available in the NCBI GEO database under access number GSE152140 (M0 versus M0Δ*tprA*) and GSE152141 (M0 versus Tn38).

### Protein purification

pLIC-*ndpA2*, pLIC-*mvaT* and pLIC-*tprA* were transformed into the *E. coli* BL21(DE3) strain. Bacteria were grown at 37°C in LB to OD_600_ ≈0.8 and protein production was induced by addition of 0.5 mM isopropyl-β-d-thiogalactopyranoside (IPTG) for 16 h at 16°C and 160 rpm. Cell pellets were resuspended in 50 mM Tris–HCl (pH 8.0), 75 mM NaCl, 1 mM ethylenediaminetetraacetic acid (EDTA) and 10 mM MgCl_2_, supplemented with 100 mg/ml DNase I, 100 mg/ml lysozyme and EDTA-free protease inhibitor (Roche). After 30 min at 4°C, resuspended cells were broken using an Emulsiflex-C5 homogenizer (Avestin) – four times at 1000 psi—and clarified by ultracentrifugation for 30 min at 20 000 *g*. Clear lysate was loaded onto 2 ml of Ni-NTA resin and then washed with 20 ml of a solution containing 50 mM Tris–HCl (pH 8.0), 75 mM NaCl and 20 mM imidazole. Proteins were eluted with the same buffer with 150 mM Imidazole as a final concentration. After His-tag TeV cleavage, the proteins were concentrated and washed in an Amicon 3 MWCO cut-off (Merck) with 15 ml Tris–HCl 50 mM (pH 8.0) and 75 mM NaCl. Based on the Bradford method, the protein concentrations were 530, 1.4 and 2 μM for TprA, MvaT and NdpA2, respectively.

### Transfer efficiency assay

The efficiency of ICE PAPI-1 transfer was evaluated as described previously (16); the experiment was carried out in triplicate. Both the PAPI-1 donor and PAO1Sm recipient strains were grown at 37°C overnight. The antibiotics used were 50 μg/ml Gm for strains carrying PAPI-1 and 500 μg/ml Cb (required for plasmid maintenance) and 2 mg/ml Sm for PAO1Sm. For plate mating, 50 μl of the donor strain was dropped on an LB agar plate and incubated at 37°C for 2 h, followed by dropping 50 μl of recipient strains on top of the donor strain. Recipients were incubated with shaking at 42°C for 2 h before mating. After incubating the mating mixture at 37°C for 8 h, it was scraped off and suspended in 1 ml of phosphate-buffered saline (PBS). Transconjugants were selected on LB agar plates containing 50 μg/ml Gm and 2 mg/ml Sm.

The number of recipients in the final mating mixtures was determined by plate counting. The transfer efficiency was calculated using the total number of transconjugants divided by the total number of recipients in the final mating mixture, as described by Carter *et al.* (15).

### Electrophoretic mobility shift assay (EMSA)

The target promoter regions were amplified from the genome of *P. aeruginosa* PA14 by PCR using the appropriate oligonucleotide pairs (see Supplementary Table S5) containing fluorescent dCTP-cy5 to allow DNA visualization. The subsequently labelled DNA (150 nM) was incubated at room temperature in a Tris–NaCl buffer (Tris 50 mM, pH 8 + NaCl 75 mM) with poly(dI-dC)•poly(dI-dC) (10 μM) and the indicated concentration of purified TprA, NdpA2 or MvaT proteins in a final volume of 13μl. After 30 min, 2 μl of loading dye (50% glycerol + 0.1% Bromophenol blue are added, then reactions are directly loaded in native gel (6% acrylamide/bis-acrylamide 37.5:1, Tris-Borate-EDTA 1X). Gels are run for 1 h at constant 45 mA. Direct DNA band visualization was performed on a Typhoon™ FLA 9500 biomolecular imager (GE Healthcare) with laser excitation at 650 nm and emission detection at 670 nm.

### SDS-PAGE and western blot

Mature PilS2 pilin expression was followed by 16.5% sodium dodecyl sulfate-polyacrylamide gel electrophoresis (SDS-PAGE) with cathode and anode buffers containing 0.1 M Tris–HCl, 0.1 M Tricine and 0.1% SDS at pH 8.25 and 0.2 M Tris–HCl at pH 8.9, respectively. Bacterial cells were grown to an OD_600_ of 0.5, induced for at least 3 h with 1 mM IPTG and collected at 0.025 OD_600_ equivalent units per μl in loading buffer. The samples were boiled for 10 min and the proteins were separated by electrophoresis before blotting onto nitrocellulose membranes. PilS2 was immunodetected using a PilS2 rabbit polyclonal antibody at a dilution of 1:150 and detected with peroxidase-conjugated goat anti-rabbit immunoglobulin G at a dilution of 1:5000 in Tris-buffered saline containing 10% non-fat milk and 0.1% Tween-20.

### Confocal biofilm analysis

Time-lapse biofilm formation was performed in flow chambers as previously described (38); each experiment was repeated two times. Briefly, the PA14_GFP_ strains tagged with green fluorescent protein (GFP) were transformed with pBBR1MCS4 (empty vector) or pBBR-*ndpA2*_*tprA* (overexpressing *ndpA2* and *tprA* genes). Bacteria were grown in M63 medium supplemented with 1 mM MgCl_2_, 0.5% casamino acids and 0.2% glucose at 30°C in flow chambers with individual channels (dimensions of 1 × 4 × 40 mm^3^). Observation was performed after 2 and 3 days with an Olympus FV-1000 microscope equipped with detectors and a filter set for monitoring GFP. The average biofilm thickness and biomass were calculated using Fiji (39). For PA14_GFP_ + pBBR1MCS4, 17 (day 2) and 23 (day 3) images have been analysed. For PA14_GFP_ + pBBR-*ndpA2*_*tprA*, 38 (day 2) and 55 (day 3) images have been analysed.

## RESULTS

### Identification of regulators controlling *ppilL2* expression

To investigate the regulation of pil2 operon expression (see Figure 1A), we monitored the expression of the *ppilL2-lacZ* fusion inserted in the PA14 strain in classical liquid conditions in rich (LB and tryptic soy broth [TSB]) and minimal medium (M9, M13 and M63). There was no significant β-galactosidase activity of the *ppilL2-lacZ* chromosomal fusion under the culture conditions tested (data not shown), suggesting that *ppilL2* expression is probably tightly regulated. We therefore investigated the regulatory genes potentially involved in *ppilL2* expression. We used the lack of detectable *ppilL2* expression after 24 h of growth on X-gal plates (no clear ‘blue’ colony on LB plates despite the presence of X-gal substrate) to carry out transposon mutagenesis using a mariner transposon. This transposon encodes a Gm resistance gene and a strong out-facing *ptac* promoter, permitting the induction of downstream genes (40). The transposon mutagenesis was performed in the PA14*_ppilL2_* strain on LB plates containing X-gal substrate to screen for blue clones. We obtained two independent libraries of 50 000 and 150 000 clones on LB medium, corresponding to a theoretic transposition efficiency of one insertion for every 130 and 43 bp, respectively. Out of the 200 000 total clones, 122 were found to be blue on plates and subjected to additional measurement of *ppilL2* expression in liquid conditions. Among these 122 clones only 30 showed, in liquid culture, *ppilL2* expression 2-fold higher than the wild-type. These 30 clones were then subjected to transposon mapping; 19 out of the 30 clones corresponded to a transposon insertion directly upstream of the *ppilL2-lacZ* fusion that led to its overexpression, among which was the Tn38 strain (see Supplementary Figure S1 and Table S3). Among the 11 other clones, transposons were mapped four times within ICE PAPI-1 in a particular DNA region ≈18.3 kb upstream of the *pil2* operon (see Figure 1A) in the strains named Tn29, Tn114, Tn115 and Tn118. Expression of the *ppilL2-lacZ* fusion was monitored in these strains after 8 h of growth and compared to the PA14*_ppilL2_* parental strain. For these four transposon mutants, the level of expression of the *ppilL2-lacZ* fusion was significantly increased compared to the parental PA14 strain (see Figure 1B). This change was accompanied by the detection of a mature form of the PilS2 protein, the pilin involved in the GI-T4SS machinery (15), which was undetectable in the PA14 strain (see Figure 1C).

These results suggest that some genes present in this DNA region are controlling *ppilL2* expression. Because Tn29, Tn114, Tn115 and Tn118 strains show the same phenotypes and based on the transposon position in these strains and the orientation of the *ptac* promoter, we hypothesized that the fusion activation is due to the overexpression of any gene downstream of the PA14_59030 gene, including the PA14_59050 to PA14_59120 genes. The putative gene functions encoded by the PA14_59000–59120 operons are listed in Supplementary Table S4. The first two genes downstream of the *PA14_59030* gene, that is, *PA14_59050* and *PA14_59060*, are strong candidates for this regulation because they respectively encode a homologue of the nucleoid-associated protein YejK family and an Arc-like DNA binding domain protein with a Ribbon–Helix–Helix (RHH) domain. For reasons discussed below, we have assigned *PA14_59050* the name *ndpa2* and *PA14_59060* the name *tprA*.

### *ndpA2* and *tprA* genes control, in synergy, *ppilL2* expression

**Figure 2.**
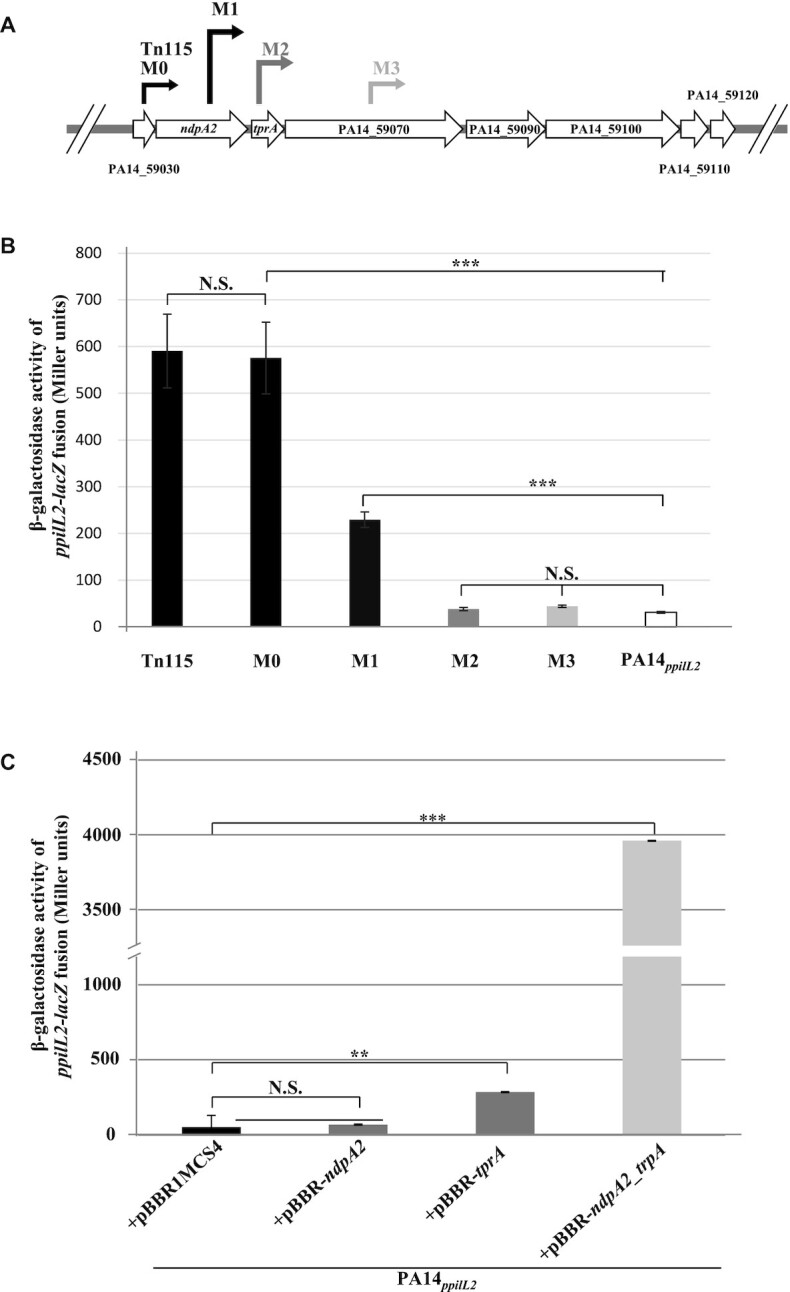
Identification of NdpA2 and TprA as regulators of *pil2* operon expression. (**A**) Position of the mariner transposon inside the PA14_59000–120 locus in Tn115, M0, M1, M2 and M3 strains. The beginning of the arrow indicates where the transposon has been inserted (the exact position is shown in Supplementary Table S3) and the tip of the arrow represents the *ptac* promoter orientation. (**B**) Expression of the chromosomal *ppilL2-lacZ* fusion, monitored in the PA14*_ppilL2_*, Tn115, M0, M1, M2 and M3 strains. (**C**) The impact of *ndpA2* or *tprA* overexpression on *ppilL2-lacZ* fusion in PA14*_ppilL2_*. For panels B and C, strains grew in LB and the *ppilL2-lacZ* fusion activities were evaluated when cells reached the early stationary phase (8 h). Data are expressed in Miller units and correspond to the mean values (with error bars) obtained from three independent experiments. Wilcoxon-Mann-Whitney tests were performed, and N.S., *, ** and *** indicate non-significant, *P* < 0.05, *P* < 0.01 and *P* < 0.001, respectively.

To locate more precisely the genes involved in *pil2* expression, we first made an allelic replacement to introduce a DNA fragment corresponding to the inserted part of the mariner transposon at different locations within the PA14_59030-PA14_59120 locus of the PA14*_ppilL2_* strain (strains M0 to M3, see Figure 2A). The insertion of this DNA fragment inside a gene disrupts its function and, thanks to the *ptac* promoter, permits the overexpression of downstream genes. The insertion of the transposon DNA into strain M0 corresponds to a re-construction of the Tn115 strain. Because strain M0 has the same *ppilL2-lacZ* expression as Tn115 (Figure 2B), it confirms the involvement of the PA14_59030-PA14_59120 locus in the regulation of *ppilL2* and rules out the possibility that secondary mutations (or other genetic events) in strain Tn115 are responsible for the activation of *ppilL2-lacZ* fusion.

Then, we evaluated the impact of these insertions on the *ppilL2-lacZ* reporter fusion (Figure 2B). The insertion of the transposon DNA in strain M1 (which disrupts the *ndpA2* gene and overexpresses the *tprA*-*PA14_59120* genes) led to a 7.4-fold induction of the transcriptional fusion. However, the induction of the *ppilL2-lacZ* fusion observed in strain M1 was 2.5 times lower than in strain M0, suggesting that the interruption of *ndpA2* modulates the expression of *ppilL2-lacZ*. More interestingly, in strain M2, where the transposon insertion occurs just after the initiation codon of the *tprA* gene, the *ppilL2-lacZ* fusion was not activated (Figure 2B). A similar result was observed in strain M3, where the transposon DNA was inserted into the *PA14_59070* gene. Taken together, these results suggest that *ndpA2* and *tprA* genes play a role in the control of the promoter of the pil2 operon.

To evaluate the contribution of the *ndpA2* and *tprA* genes on the *ppilL2-lacZ* fusion, each gene was cloned into the replicative vector pBBR1MCS4 (see Materials and methods) and introduced in the PA14*_ppilL2_* strain to monitor the impact of their overexpression on the expression of the *ppilL2-lacZ* fusion. As shown in Figure 2C, *tprA* overexpression induced the fusion 5.3-fold, while *ndpA2* overexpression had no significant impact (1.3-fold induction). Interestingly the induction of the fusion triggered by *tprA* overexpression was lower than in strain M0, suggesting that a second factor could be involved in the regulatory mechanism. Because the inactivation of *ndpa2* in M1 reduces the expression of *ppilL2-lacZ* (Figure 2B), we cloned *ndpA2* and *tprA* genes together in pBBR1MCS4 (pBBR-*ndpA2_tprA*). This construct was introduced by mating in strain PA14*_ppilL2_* to monitor the *ppilL2-lacZ* fusion expression (Figure 2C). Interestingly, the co-expression of *ndpA2* and *tprA* induced the expression of the *ppilL2-lacZ* fusion higher than *tprA* alone or strain M0 (74-fold induction).

Taken together, these results show that the *tprA* induces the *ppilL2* promoter, with an enhanced action when *ndpA2* is co-expressed. To confirm the exclusive contribution of *ndpA2* and *tprA* genes on the *ppilL2* promoter, the *ppilL2-lacZ* fusion was introduced into *P. aeruginosa* PAO1 strain lacking PAPI-1 and PAPI-2. Then, the PAO1*_ppilL2_* strain was transformed with pBBR1MCS4, pBBR-*ndpA2*, pBBR-*tprA* or pBBR-*ndpA2_tprA* and the *ppilL2-lacZ* fusion was monitored after 8 h of growth in LB. As shown in Supplementary Figure S2, the *ppilL2-lacZ* followed the same expression profile as in PA14, confirming that, besides *ndpA2* and *tprA*, no other PA14-specific genes are involved in the *ppilL2* promoter regulation.

### TprA directly binds the promoter of the *pil2* operon

**Figure 3.**
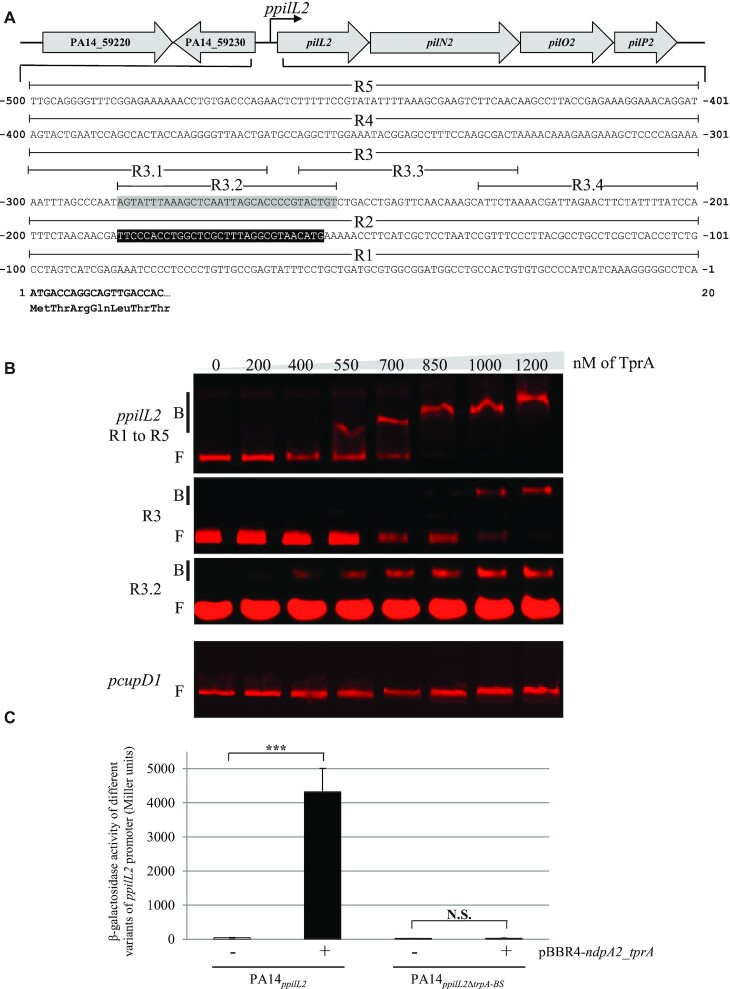
TprA binds the *ppilL2* promoter. (**A**) Promoter region of the *pilL2* gene locus in the PA14 strain. The *ppilL2* promoter predicted by Carter and colleagues (12) is indicated by a black box. The gray-boxed sequence from − 254 to − 287 indicates the TprA binding site (*tprA-BS)*. The *pilL2* coding sequence is in bold. (**B**) EMSA was performed with purified TprA protein, at concentrations ranging from 0 to 1200 nM, and different regions of *ppilL2* or *pcupD1* promoter (negative control). Retarded nucleoprotein complexes were identified by B (Bound); free DNA is indicated by F (Free). (**C**) Expression of the chromosomal *ppilL2-lacZ* and *ppilL2_Δ__tprA-BS_-lacZ* fusions monitored in PA14*_ppilL2_*+ pBBR1MC4 or PA14*_ppilL2_* +pBBR4-*ndpa2*_*tprA*. The strains grew in LB and the expression of two fusions were evaluated when cells reached the early stationary phase (8 h). Data are expressed in Miller units and correspond to the mean values (with error bars) obtained from three independent experiments. Wilcoxon–Mann–Whitney tests were performed and N.S. and *** indicate non-significant and *P* < 0.001, respectively.

We investigated whether the *pil2* operon regulation involves the direct binding of TprA to the *ppilL2* promoter. The DNA region encompassing the *ppilL2* promoter (Figure 3A) was amplified to generate a 520 bp DNA fragment and TprA protein was purified for EMSA. Retarded DNA/protein complexes were observed for TprA amounts higher than 550 nM (see Figure 3B). As a negative control, a 500 bp DNA fragment encompassing the promoter region of the *cupD1* locus belonging to the PAPI-1 genomic island was amplified and tested for its ability to bind TprA. The *cupD1* promoter (*pcupD1*) was chosen because its expression is not controlled by TprA (see below). As show in Figure 3B, TprA was unable to bind the p*cupD1* promoter region, whereas it directly controlled the expression of the *pilL2* gene.

To localize the TprA binding site, the DNA region encompassing the *ppilL2* promoter was divided into five parts, each approximately 100 bp, named R1 to R5 (see Figure 3A). These five regions were obtained hybridizing forward Cy5-labelled single strand DNA (ssDNA) and reverse ssDNA (see Supplementary Table S5) and subjected to EMSA with 0 and 1 μM TprA. As show in Supplementary Figure S3, this protein only bound to the R3 region. To identify the TprA binding site more precisely, this R3 region was subdivided into four parts of 33 bp named R3.1 to R3.4 (Figure 3A). Each part was assayed for its capacity to interact with TprA by EMSA. TprA only bound to the R3.2 sub-region (Figure 3B). This R3.2 sub-region was called the TprA binding site (*tprA-BS*). To confirm the role of this sub-region in the regulation of the *ppilL2* promoter, we constructed a chromosomal *ppilL2-lacZ* reporter fusion with the tprA-BS region deleted (*ppilL2_Δ__tprA-BS_-lacZ*) and inserted it into the PA14 strain (PA14*_ppilL2_*_Δ_*_tprA-BS_-lacZ*). We then compared expression of this fusion to the *ppilL2-lacZ* fusion when we co-overexpressed *ndpA2* and *tprA* genes (pBBR-*ndpA2*_*tprA*, Figure 3C). *tprA-BS* deletion abolished *ppilL2* promoter activation by the NdpA2-TprA couple. This result confirms the crucial role of this region in *pil2* operon regulation.

### MvaT directly silences the *ppilL2* promoter

We previously demonstrated in this study that NdpA2 enhances the action of TprA. Bioinformatic predictions suggest that NdpA2 could be an anti-histone-like nucleoid structuring (H-NS) protein due to its similarity with the anti-H-NS NdpA (PA14_14220). Thus, we performed directed mutagenesis analyses to identify which H-NS protein(s) NdpA2 could counteract.

**Figure 4.**
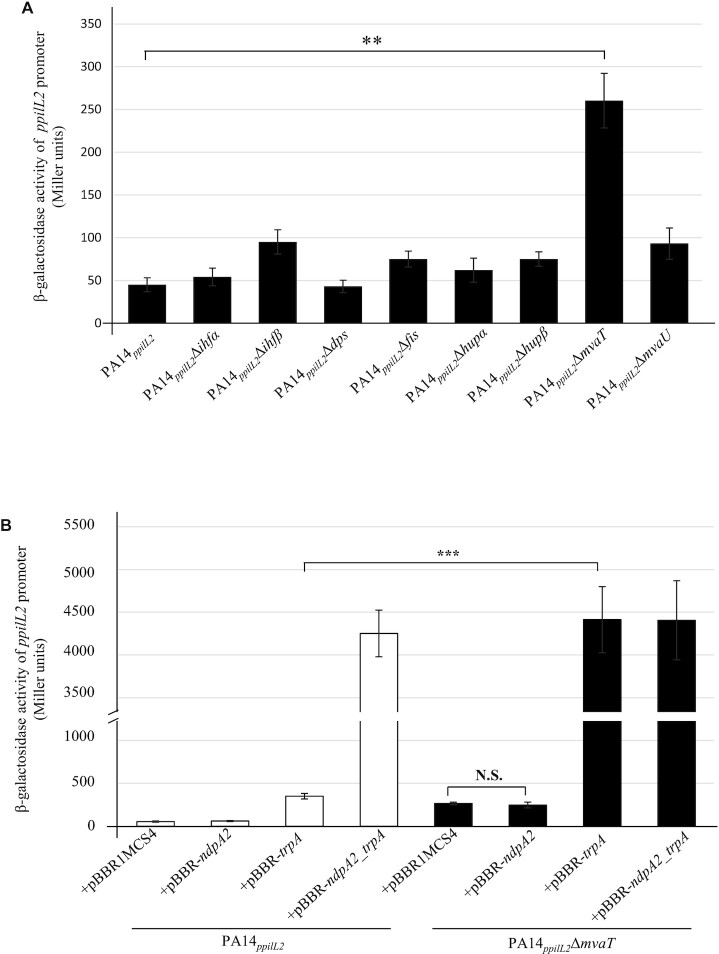
Role of H-NS proteins on *pilL2* expression. (**A**) Expression of the chromosomal *ppilL2-lacZ* fusion monitored in various PA14*_ppilL2_* strains. (**B**) Activity of the transcriptional chromosomal fusions were monitored after 8 h of growth in PA14*_ppilL2_* or PA14*_ppilL2_ΔmvaT* strains overexpressing or not *ndpA2* and/or *tprA* genes. Data in Miller units correspond to the mean values obtained from three independent experiments. Wilcoxon–Mann–Whitney tests were performed and N.S. and *** indicate non-significant and *P* < 0.001, respectively.

The *P. aeruginosa* PA14 genome contains eight major H-NS proteins: IHFα, IHFβ, Dps, Fis, HUα, HUβ, MvaT and MvaU (41–43). We therefore inactivated each of the PA14 H-NS proteins and monitored their impact on the *ppilL2-lacZ* reporter fusion. (Figure 4A). Only *mvaT* gene inactivation induced the *ppilL2-lacZ* reporter. This indicates that among the tested H-NS-like proteins, only MvaT represses *ppilL2* expression.

To confirm that no other H-NS protein is involved in *ppilL2* promoter repression, we overexpressed the *tprA* gene in each of the H-NS mutants. As shown in Supplementary Figure S4, when *tprA* is overexpressed the *ppilL2-lacZ* fusion showed a strong expression in the PA14*_ppilL2_*Δ*mvaT* strain (∼2100 Miller units). In the other strains, the overexpression of *tprA* only induced the fusion at a level similar to the PA14*_ppilL2_* strain (∼680 Miller units). This suggests that MvaT is the only HN-S protein involved in the regulation of *ppilL2* and it acts as a repressor on it.

To further characterize the role of MvaT in *pil2* regulation, we overexpressed *ndpA2* or *tprA* in the PA14*_ppilL2_*ΔmvaT strain and compared ppilL2-lacZ activations to those in PA14*_ppilL2_* (Figure 4B). In the PA14*_ppilL2_*ΔmvaT strain, ndpA2 overexpression alone did not induce the fusion. However, tprA overexpression strongly induced this fusion at a comparable level to the PA14*_ppilL2_* strain overexpressing *ndpA2* and *tprA* (pBBR-*ndpA2_tprA*). This latter result suggests that the absence of MvaT allows TprA to induce *ppilL2-lacZ* fusion expression at its observed maximal level without the requirement of NdpA2.

**Figure 5.**
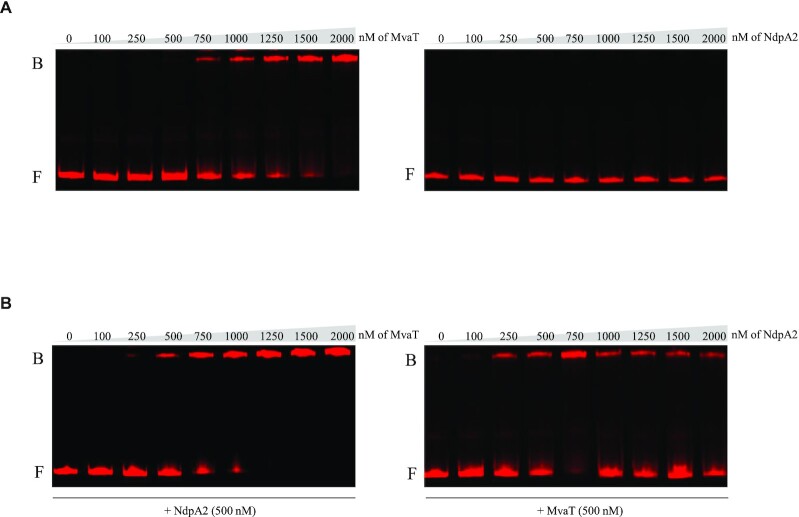
MvaT binds the *ppilL2* promoter and NdpA2 increases its affinity for DNA. (**A**) EMSA was performed with purified MvaT (left panel) or NdpA2 (right panel) proteins, at concentrations ranging from 0 to 2000 nM, and the 500 bp *ppilL2* promoter. (**B**) In the left panel, EMSA was performed with the 500 bp *ppilL2* promoter region, 500 nM of NdpA2 and different concentrations of MvaT ranging from 0 to 2000 nM. In the right panel, EMSA was performed with the 500 bp *ppilL2* promoter region, 500 nM of MvaT and different concentrations of NdpA2 ranging from 0 to 2000 nM. Retarded nucleoprotein complexes are identified by B (bound); free DNA is indicated by F (free).

To better characterize how MvaT and NdpA2 influence the expression of *ppilL2*, we used EMSA to test the ability of MvaT and NdpA2 to bind the *ppilL2* promoter. MvaT and NdpA2 were purified and then incubated at different concentrations with the 500 bp DNA region encompassing the *ppilL2* promoter (Figure 5A). Retarded DNA/protein complexes were observed for MvaT ≥750 nM (Figure 5A, left panel) whereas no DNA/protein complexes were observed for NdpA2 (Figure 5A, right panel). Because in the PA14*_ppilL2_*Δ*mvaT* strain *tprA* overexpression is enough to trigger the *ppilL2-lacZ* fusion at a comparable level to the PA14*_ppilL2_* strain overexpressing *ndpA2* and *tprA* (pBBR-*ndpA2_tprA*), we hypothesized that NdpA2 may prevents or drives out MvaT binding. To test this hypothesis, we incubated the DNA region encompassing the 500 bp *ppilL2* promoter region with different concentrations of MvaT and a NdpA2 concentration of 500 nM (Figure 5B, left panel). Surprisingly, NdpA2 addition did not prevent or drive out MvaT binding; on the contrary, it increased the affinity of MvaT affinity for the *ppilL2* promoter. Indeed, when NdpA2 was added at 500 nM in the reaction, MvaT could bind to the *ppilL2* promoter region at a concentration at 250 nM, a concentration three-times lower than when no NdpA2 is added. To confirm that NdpA2 increases the affinity of MvaT for the *ppilL2* promoter, we incubated this promoter with a fixed concentration of 500 nM MvaT and different concentrations of NdpA2 (Figure 5B, right panel). In this experience, if we did not observe any DNA-binding of NdpA2 without MvaT addition, a gel shift is observed when a concentration >250 nM of NdpA2 is added to the reaction.

Taken together, these results show that MvaT H-NS silences the *ppilL2* promoter by directly binding it and this binding reduces the capacity of TprA to induce *ppilL2* expression. NdpA2 counters the repressive action of MvaT by increasing its affinity for DNA, which probably has the effect of modifying the local DNA supercoiling (see Discussion).

### TprA is major activator of ICE PAPI-1 genes

**Figure 6.**
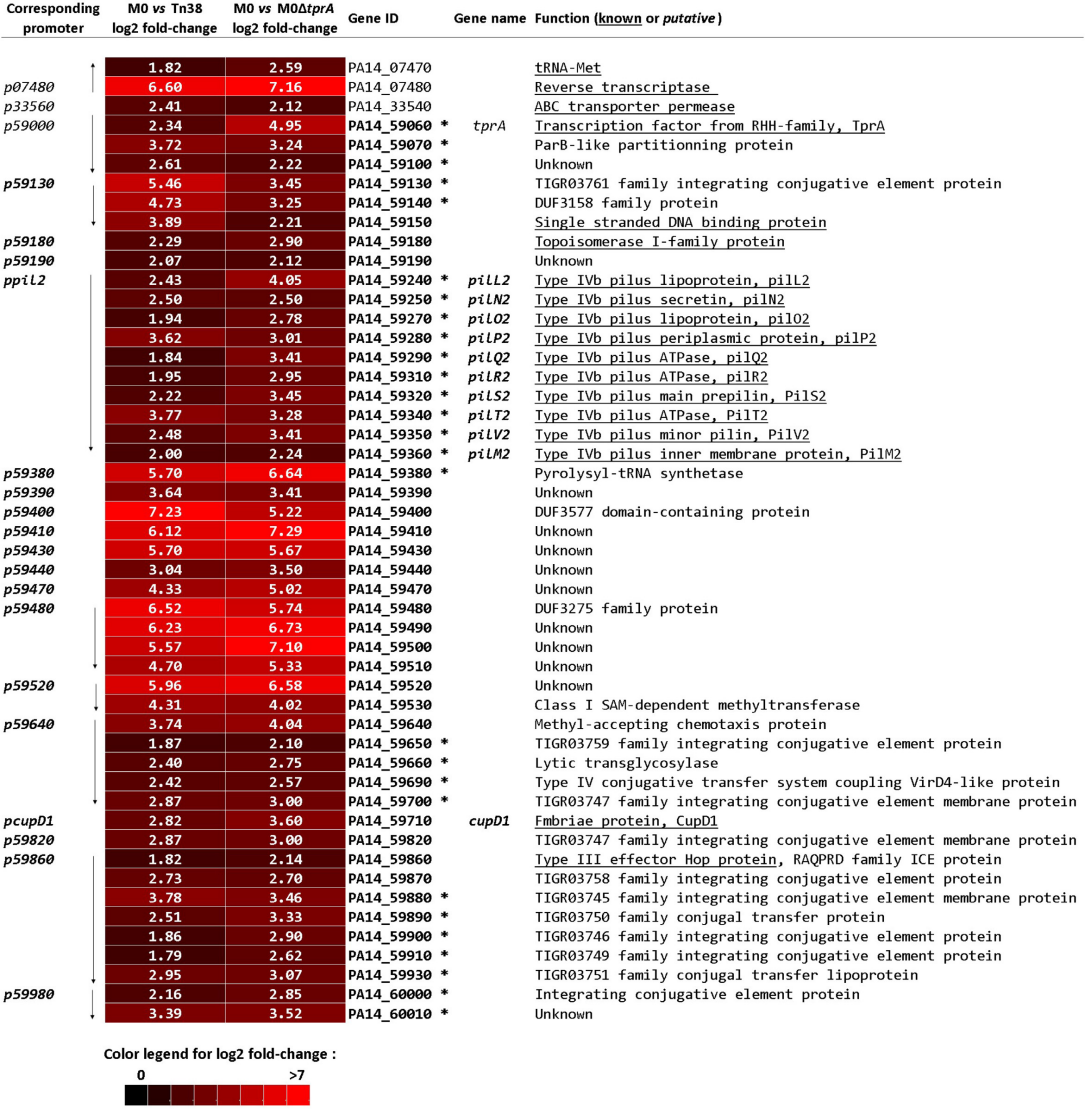
Genes differentially regulated by *ndpA2* and *tprA* overexpression (M0 strain) versus non-overexpressing strain (Tn38 strain) or versus the strain with inactivated *tprA* gene (M0Δ*tprA* strain). The colour chart, at the bottom of the figure, illustrates the expression level of each gene based on an average of two experiments (see Materials and Methods). Red indicates the induced genes in M0 according to the colour chart. The log2fold-change in mRNA levels (M0/Tn38; column 2) or (M0/M0Δ*tprA*; column 3) are indicated for each gene by the white number in the boxes. Genes are identified by their unique ID (Gene ID) and by their gene name. Genes are listed in chromosomal order to illustrate the transcriptional units. The transcription direction is indicated by an arrow. Genes in bold are located within ICE PAPI-1 and essential genes for PAPI-1 transfer (11,12) are indicated by an *. The function of the genes, known (underlined) or putative, are provided in column 6.

To further characterize the impact of the TprA regulator on the PA14 genome, we carried out transcriptomic experiments using MGPA arrays (38). RNA was extracted from M0, Tn38 and M0Δ*tprA* strains after 7 h grown in LB at 37°C. We compared the transcriptomic profile of the M0 strain with the transcriptomic profile of the Tn38 (M0/Tn38) or M0Δ*tprA* strains, in which the *tprA* gene is inactivated by insertion of a stop codon just after its start codon (M0/M0*ΔtprA*). In the M0/Tn38 experiment, we compared the RNA expression profile to the M0 strain, in which the *ndpA2*-*PA14_59120* locus is induced by the mariner transposon to the Tn38 control strain, where this same transposon is inserted just before the transcriptional fusion *ppilL2-lacZ* on the core genome. Applying selective criteria, we found 74 differentially expressed genes (Supplementary Figure S5). To determine which of them were under TprA control, we compared the RNA expression profile of strain M0 to strain M0*ΔtprA* (M0/M0*ΔtprA*). Here, we found 71 differentially expressed genes (see Supplementary Figure S6). To focus on genes belonging to the TprA regulon, genes similarly expressed in these two sets of experiments were selected, and 50 genes were identified as being positively induced by TprA (Figure 6). All those genes were organized in 21 transcriptional units; one noticeable observation is that 47 of these genes belong to the PAPI-1 genomic island and 27 of them have been previously described to be essential for PAPI transfer (7). Interestingly, we found the genes belonging to the *pil2* locus, validating our approach, but also genes encoding conjugation associated proteins and genes described to be involved in DNA mobilization, integration and partition activities (see Figure 6). The remaining genes are mainly of unknown function, but several have been described as important for virulence in a mouse thermal injury model, such as *PA14_59870*, *PA14_59490* or *PA14_59150* (7,13,14). These results indicate that TprA is a main regulator of PAPI-1 transfer but could also be involved in the *P. aeruginosa* PA14 pathogenicity.

Our transcriptome results also showed that three genes outside of PAPI-1 and distributed into two different loci are regulated by TprA. These loci are an ABC transporter permease (*PA14_33540*) and a putative reverse transcriptase (*PA14_07480*) in an operon with a tRNA-Met (*PA14_07470*). The *PA14_07480* gene was most strongly regulated by TprA, with a 95-fold change (log2 fold-change ≈ 7).

### Validation of differentially expressed transcriptional units

**Figure 7.**
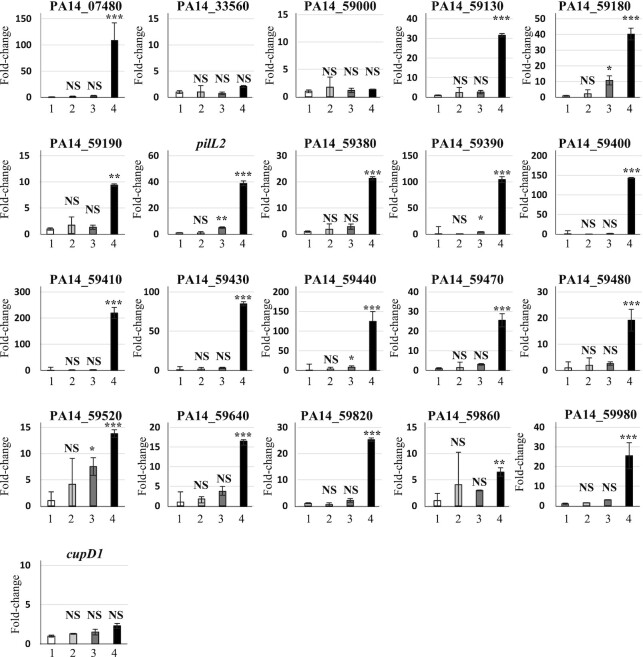
Validation of differentially expressed genes by TprA and NdpA2. qRT-PCR were performed in triplicate (see Materials and Methods) on the first gene of each transcription unit detected as being differentially expressed by our microarray experiments (see Figure 6). The identifies are 1: PA14 + pBBR1MCS4, 2: PA14 + pBBR-*ndpA2*, 3:PA14 + pBBR-*tprA*, 4: PA14 + pBBR-*ndpA2_tprA*. The Y-axis represents the relative fold change between PA14 + pBBR1MCS4 and the other strains. Data were analysed using REST software in which significant differences are determined by an ANOVA using approximate tests (34). *, **, *** and NS indicate *P* < 0.05, *P* < 0.01 and *P* < 0.001 and a non-significant difference, respectively.

The transcriptional units found to be differentially regulated in transcriptomic experiments were further analysed by qRT-PCR to determine whether their regulation also requires the synergic action of NdpA2 and TprA. Total RNA from PA14 + pBBR1MCS4 and from PA14 harbouring pBBR-*ndpA2*, pBBR-*tprA* or pBBR-*ndpA2_tprA* plasmids were extracted, reverse transcribed into cDNA and the expression of the 21 transcriptional units were assessed by qRT-PCR (Figure 7). The obtained results confirmed that 18 of 21 are controlled by the synergic action NdpA2 and TprA. However, they displayed different dependency on these regulators. Thirteen were exclusively induced when both regulators are co-expressed (*PA14_07480*, *PA14_59130*, *PA14_59190*, *PA14_59380*, *PA14_59400*, *PA14_59410*, *PA14_59430*, *PA14_59470*, *PA14_59480*, *PA14_59640*, *PA14_59820*, *PA14_59860* and *PA14_59980*; see Figure 7). The other units were less dependent on these regulators’ synergic action and could be slightly induced by TprA alone. However, their expression was always higher when both regulators are co-produced. These results also showed that *PA14_33560*, *PA14_59000* and *cupD1* genes are not controlled by NdpA2 and TprA, suggesting that these three genes belong to the 5% false positive generated by our transcriptomic analysis pipeline.

To determine which of the 21 promoters are directly controlled by TprA, we conducted EMSA experiments between these promoters and TprA (see Supplementary Figure S7). We observed that TprA directly controlled 13 of the 21 promoters, including the *pil2* operon, *PA14_59380* gene and the *PA14_59640–59700*, *PA14_59860–59960*, *PA14_59980–60020* operons involved in PAPI-1 transfer and PA14_59520–59530 involved in PA14 pathogenicity (see Supplementary Figure S7) (7,15).

Taken together, these results confirm that TprA, with the synergic action of NdpA2, is a main regulator of PAPI-1 transcriptional units, exerting a direct and positive control on 13 of them and an indirect induction on 5 of them.

### NdpA2/TprA dependent phenotypes

Our transcriptomic data revealed that NdpA2 and TprA not only control the expression and biosynthesis of GI-T4SS, but also the expression of essential genes for PAPI transfer, suggesting that the couple NdpA2/TprA could be the main regulator of the PAPI-1 transfer. To validate this hypothesis, we performed a transfer assay on PA14 strains overexpressing *ndpA2, tprA* or both genes. As shown in Table 1, *ndpA2* gene overexpression did not significantly induced ICE PAPI-1 transfer, whereas *tprA* gene overexpression induces it 8.7 fold (compared to the control strain PA14 + pBBR1MCS4 or PA14). Interestingly, when both regulators are overexpressed, ICE PAPI-1 transfer was increased more than 1 × 10^3^ times, confirming the role of NdpA2 and TprA in ICE PAPI-1 transfer. To strengthen our results, we also examined the impact of *ndpA2* or *tprA* inactivation in the M0 strain on PAPI-1 transfer. As shown in Table 1, *ndpA2* inactivation reduced the ICE transfer by 40-fold whereas *tprA* inactivation reduced it more than 1100-fold.

**Table 1.** Impact of *ndpA2* and *tprA* expression on ICE PAPI-1 transfer

Donor strain	Transfer efficiency (number of transconjugants/recipient)
PA14	9.6 × 10^−8^ ± 0.75 × 10^−8^
M0	5.9 × 10^−5^ ± 0.24 × 10^−5^
M0Δ*ndpa2*	1.4 × 10^−6^ ± 0.26 × 10^−6^
M0Δ*tprA*	5.1 × 10^−8^ ± 0.44 × 10^−8^
PA14 + pBBR1MCS4	9.9 × 10^−8^ ± 1.23 × 10^−8^
PA14 + pBBR-*ndpA2*	1.2 × 10^−7^ ± 0.96 × 10^−7^
PA14 + pBBR-*tprA*	8.6 × 10^−7^ ± 1.04 × 10^−7^
PA14 + pBBR-*ndpA2*_*tprA*	1.4 × 10^−4^ ± 0.53 × 10^−4^

**Figure 8.**
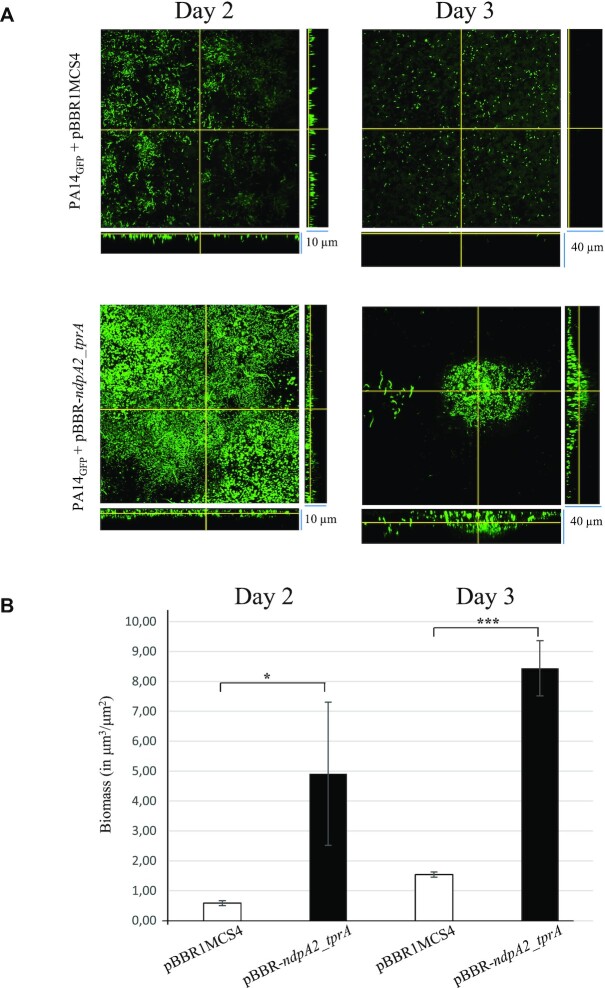
*ndpA2* and *tprA* overexpression induce biofilm formation. (**A**) Biofilm formation of PA14_GFP_ + pBBR1MCS4 and PA14_GFP_+ pBBR-*ndpa2_tprA* strains monitored at days 2 and 3. Stacked confocal scanning laser microscopy images of biofilms and corresponding extracted z images and their respective xy and xz planes are presented. (**B**) Biofilm biomass of PA14_GFP_ + pBBR1MCS4 and PA14_GFP_+ pBBR-*ndpa2_tprA* strains monitored at days 2 and 3. The biomass, expressed in μm^3^/μm^2^, corresponds to the mean values obtained from two independent experiments. Wilcoxon–Mann–Whitney tests were performed and * and *** indicate *P* < 0.05 and *P* < 0.001, respectively. The experiment was repeated twice, independently, and the images presented correspond to the typical results observed in both experiments.

Previous studies have shown that the acquisition of PAPI-1 by *P. aeruginosa* promotes biofilm formation (14). To determine the impact of the NdpA2/TprA couple on biofilm formation, we monitored its formation in flow cell using confocal microscopy (Figure 8).

To visualize the bacteria inside the biofilm, the PA14_GFP_ strain expressing the green fluorescent protein was transformed with the empty vector (pBBR1MCS4) or the pBBR-*ndpA2_tprA* plasmid (Figure 8). After 2 days of development, the biofilm formed by PA14_GFP_ with the empty vector or the pBBR-*ndpA2_tprA* plasmid was a monolayer; however, the biomass of the strain with the pBBR-*ndpA2_tprA* was 8.3 times greater than the one with the empty vector (Figure 8, Day 2). After 3 days, the PA14_GFP_ overexpressing *ndpA2* and *tprA* displayed a biofilm phenotype, allowing cells to form mushrooms with a prominent three-dimensional expansion (average thickness of 36 μm). At the same time scale, the PA14_GFP_ strain carrying the empty vector (pBBR1MCS4) formed only a multi-layered cell carpet with a tightness of 4 μm in average on the glass substrate. Moreover, on day 3, the biomass of PA14_GFP_ with the pBBR-*ndpA2_tprA* was 5.5 times greater than the one with the empty vector (Figure 8, Day 3).

Taken together, these results show that Ndpa2/TprA activation triggers PAPI-1 transfer, which is associated with a biofilm formation phenotype.

## DISCUSSION

HGT has been recognized as one of the main contributors to bacterial evolution and diversification. Through a one-step acquisition of genetic materials, bacteria gain new functions, enabling them to colonize various environments. *P. aeruginosa* is well known for its plastic genome composed of core genes and a variable number of horizontally acquired genes. ICE PAPI-1 is one of the largest and the main pathogenicity genomic islands in *P. aeruginosa*. Its transfer, through a conjugative pilus encoded by the *pil2* operon, is tightly regulated to maintain its quiescence during growth. Carter and colleagues showed that the expression of *ppilL2* (*pil2* main promoter) increases in the stationary growth phase and suggested that ICE PAPI-1 transfer could be regulated by a stationary-phase regulator belonging to the core genome (15).

In this study, we showed that a high expression of the *pil2* operon is related to a marked increase in PAPI-1 transfer. In accordance with their function or homology, PA14_59050 has been renamed NdpA2 due to its strong similarity (97% identity) with another nucleoid-associated protein of PA14: NdpA (PA14_14220) and PA14_59060 has been renamed TprA for (Transfer of PAPI-1 regulator A). TprA is a regulator belonging to the very large family of RHH transcription factors. This family comprise many regulators that play important roles in plasmid transfer by conjugation, including TraY, TprM (Plasmid F), TrwA (Plasmid R388), NikA (Plamsid R64), TraK (Plasmid Rp4) and MobC (Plasmid RSF1010) (48–52). While the RHH regulators are well described to play a role in plasmid transfer, to our knowledge, TprA is the first RHH regulator identified to be involved in an ICE transfer. This observation reinforces the idea developed by Carter and collaborators that the ancestor of ICE PAPI-1 is a plasmid, likely the pKLC102 plasmid (15).

Moreover, we demonstrated that the regulation of the *pil2* operon involves a regulator encoded on the core genome and two regulators encoded within the ICE. In this regulatory mechanism MvaT, an H-NS encoded within the core genome, represses the PAPI-1 conjugative pilus biosynthesis, and this repression can be released by NdpA2, a nucleotide-associated protein encoded within ICE PAPI-1 that modifies the binding of MvaT on the conjugative pilus *ppilL2* promoter. MvaT is one of the most abundant DNA-binding proteins of *P. aeruginosa*. As a nucleoid-associated protein, it plays a role in the physical organization of chromosomal DNA and it is a prominent global regulator of gene expression (44). The mechanism by which MvaT performs its functions is based on its interaction with DNA. It has been shown that MvaT can form both dimers and higher‐order oligomers (45,46). MvaT can bridge double-stranded DNA molecule to form hairpin structures, or when MvaT increases its DNA affinity or concentration, it can polymerize along the double-stranded DNA causing DNA stiffening. This DNA stiffening structure is a formation of a rigid MvaT protein filament along the DNA that reorganizes the DNA supercoiling in its vicinity (45). In many organisms, these two binding modes can be switched by adjusting the magnesium concentration (over a physiological range) as well as the pH and temperature (47). However, MvaT nucleoprotein filaments have been reported to be insensitive to changes in salt osmolarity, pH and temperature, and are thus distinct from those formed by H-NS proteins in other organisms (45). Here, we demonstrated that MvaT can bind the *ppilL2* promoter and that its affinity for DNA is increased in the presence of NpdA2. A plausible regulatory mechanism could be proposed in which, when *ndpA2* and *tprA* are not expressed, MvaT binds to the *ppilL2* promoter and represses its expression. When *ndpA2* and *tprA* expression is induced, in response to an unknown signal, NdpA2 increases the affinity of MvaT for DNA and promotes a switch between MvaT dimers and higher‐order oligomers. This switch triggers a local DNA reorganization that allows TprA to bind to the promoter. In this mechanism, TprA is the key regulator because its deletion abolishes the expression of the *ppilL2* promoter, whereas NdpA2 and MvaT only tune its expression. NdpA2 and TprA are the two major contributors coded by PAPI-1 on the *ppilL2* regulation because their overexpression in the PAO1 strain, which lacks PAPI-1 and PAPI-2, presents the same regulatory profile. Nevertheless, the *ppilL2* expression level is lower in the PAO1 strain than in the PA14 strain, suggesting that other regulatory phenomena could be involved.

With our transcriptomic approach, we showed that TprA is also a main regulator of PAPI-1-encoded genes. Indeed, TprA controls the expression of several PAPI-1 transcriptional units. Among them, we found many genes associated with DNA transfer. Our results revealed that TprA controls three genes localized outside of PAPI-1. Among them, *PA14_07480* encodes a putative reverse transcriptase and is the top ranked differentially expressed gene when *tprA* is overexpressed. Of note, its role in the context of ICE PAPI-1 transfer or *P. aeruginosa* PA14 pathogenicity is still unknown. Using qRT-PCR, we showed that most of the genes controlled by TprA are fully induced when NdpA2 is also co-expressed. The NdpA2 requirement for maximal induction of these PAPI-1 genes by TprA suggests that these genes could also be repressed by MvaT. Thus, MvaT appears to be a global repressor of PAPI-1 encoded genes and, perhaps participates in a phenomenon known as xenogeneic silencing (also called exosilencing), whose objective is to minimize the negative impact of genes acquired by HGT on bacterial fitness (53,54). Indeed, although HGT plays a major role in microbial evolution, foreign sequences could have a positive or negative impact on bacterial fitness (53). To minimize the negative impacts, bacteria can repress the transcription of these foreign genes through H-NS proteins which are important repressors of transcription in Gram-negative bacteria. For example, a constitutive expression of the genes encoded by the ICE ICE*clc* from *Pseudomonas* sp. strain B13 has been shown to cause a growth reduction leading to the death of the cell host (21). This deleterious impact on bacterial survival explains the observed thin regulation of ICE*clc* transfer and reinforces the need for an organism to exosilence genes carried by exogenous DNA, because they can be poisoned gifts. To assess whether PAPI-1 activation also leads to massive cell hosts death, we compared the survival rate of M0 to M0Δ*tprA* in stationary phase. We observed a slight 2.6-fold reduction in M0 survival (data not shown). This result is very different from what was observed for ICE*clc* activation, which leads to massive population death with a loss of two orders of magnitude in viability (21). Thus, in the case of *P. aeruginosa*, activation of ICE PAPI-1 does not appear to be a significant threat to its survival. Nevertheless, this point needs to be addressed more fully in future studies.

Another interesting feature resulting from TprA/NdpA2 activation is the formation of a specific biofilm by PA14. Indeed, this biofilm presents an atypical gene signature. *P. aeruginosa* has around 24 systems involved in biofilm construction in its genome, including fimbriae, pilus, exopolysaccharide, flagella and adhesin (55). Our results demonstrated that among these 24 systems, the TprA/NdpA2 pathway only induces the Pil2 system and in this way differs greatly compared to classical biofilm induction by other signalling pathways (i.e. the GacS/GacA pathway, the PprB/PprA pathway or the AmrZ transcription factor). Using transmission electron microscopy, Carter and collaborators showed that the Pil2 pilus form long, thin, and bundled pili and have a twisted appearance all around the cell surface. Thus, we suggest that the atypical biofilm formed by the TprA/NdpA2 activation has the purpose of permitting cell–cell contact during conjugation and the formation of mating pairs to facilitate ICE PAPI-1 transfer.

In this work, we have unraveled the molecular regulatory pathway controlling PAPI-1 transfer in *P. aeruginosa*, with a tripartite relationship between TprA, NdpA2 and MvaT. TprA belongs to the RHH protein family; these kinds of proteins share the same fold, but their activation mechanisms are diverse. Some are activated by small compounds, for example, in *E. coli*, NikR is activated by binding Ni^2+^ ions and MetJ is activated by an interaction with *S*-adenosylmethionine (56). Other RHH proteins are activated by protein–protein interactions, such as FitA of *Neisseria gonorrhoeae*, which forms a complex with FitB to bind DNA (57). In the case of TprA, the signal activation the system is still unknown, and its identification may lead in the future to the development of new strategies to limit *P. aeruginosa* virulence or the spread of ICE PAPI-1 in *P. aeruginosa* populations.

## DATA AVAILABILITY

The microarray dataset is available in the NCBI GEO database under access number GSE152140 (M0 vs M0Δ*tprA*) and GSE152141 (M0 vs Tn38).

## Supplementary Material

gkab827_Supplemental_Files
