# Supplementary material for: The horizontal transfer of Pseudomonas aeruginosa PA14 ICE PAPI-1 is controlled by a transcriptional triad between TprA, NdpA2 and MvaT
Source: Nucleic Acids Res. 2021 Oct 13;49(19):10956–74. doi: 10.1093/nar/gkab827 (PMC8565334; doi:10.1093/nar/gkab827)
Supplement: gkab827_Supplemental_Files [file gkab827_supplemental_files.zip › Dangla-Pelissier_et_al-Supplementary_material_R2.docx]

Supplementary Information for

**The horizontal transfer of** P. aeruginosa **PA14 ICE PAPI-1 is controlled
by a transcriptional triad between TprA, NdpA2 and MvaT**

Gauthier Dangla-Pélissier^1^, Nicolas Roux^1^, Victoria Schmidt^1^, Gaël Chambonnier^1^, Moly Ba^1^, Corinne Sebban-Kreuzer^1^, Sophie de Bentzmann^1^, Caroline Giraud^2^ and Christophe Bordi^1*^

1 LISM, IMM, Aix-Marseille University, Marseille 13402, France

2 U2RM Stress/Virulence, Normandy University, UNICAEN, 14000, Caen, France

*To whom correspondence should be addressed. Email: [bordi@imm.cnrs.fr](mailto:bordi@imm.cnrs.fr)

**This PDF file includes:**

Figures S1 to S7

Tables S1 to S5

Supplementary references

**Figure S1. Expression of the *ppilL2–lacZ* chromosomal** **in transposed PA14 strains**. Each transposon (Tn) strain was grown in LB and the *ppilL2-lacZ* fusion was evaluated when cells reached the early stationary phase (7 h). Data are expressed in Miller units and correspond to the mean values (with error bars) obtained from three independent experiments. Black columns represent Tn strains with mariner transposon inserted before *ppilL2-lacZ* fusion. Grey columns represent Tn strains with mariner transposon inserted within ICE PAPI-1. White columns represent Tn strains with mariner transposon inserted within PA14 core genome.

**Figure S2. Impact of *ndpA2* and/or *tprA* overexpression on *ppilL2-lacZ* fusion in PAO1*_ppilL2_*.** The strains growth in LB and the *ppilL2-lacZ* fusion activities were evaluated when cells reached the early stationary phase (8 h). Data are expressed in Miller units and correspond to the mean values (with error bars) obtained from three independent experiments. Wilcoxon–Mann–Whitney tests were performed, and N.S., *, ** and *** indicate non-significant, p<0.05, p<0.01 and p<0.001, respectively.

**Figure S3. Identification of TprA binding site within *ppilL2* promoter.** The 500 bp region upstream the *pilL2* gene was divided into 100 bp fragments called R1 to R5 (see Figure 3) and tested by EMSA with 0 µM (upper panel) or 1 µM (lower panel) of purified TprA protein (see Materials and Methods). B: bound DNA; F: free DNA.

**Figure S4. Expression of the *ppilL2–lacZ* chromosomal fusion** **in various PA14 strains overexpressing pBBR-*tprA*.** The PA14 strains carrying *ppilL2–lacZ* fusion where conjugated with the pBBR-*tprA* plasmid and the fusion activity was recorded after 7 h of growth in LB. Corresponding β-galactosidase activities are expressed in Miller units and correspond to mean values (with error bars) obtained from three independent experiments. Wilcoxon–Mann–Whitney tests were performed, ** and NS indicate p<0.01 and non-significant, respectively.

**Figure S5. Differentially expressed genes identified from genome-wide transcription profiling following comparison of M0 versus Tn38 strains.** The colour chart illustrates the expression level of each gene based on an average of two experiments (see Materials and Methods). Red indicates the induced genes, and green indicates the genes repressed in the M0 strain. The log2fold-changes in mRNA levels (M0/Tn38) are indicated for each gene by the number in the boxes. Genes are identified by their unique ID (Gene ID) and are listed in chromosomal order to illustrate the transcriptional units. Genes in bold are located within ICE PAPI-1. The function of genes, known (underlined) or putative, are provided in column 3.

**Figure S6.** **Differentially expressed genes identified from genome-wide transcription profiling following comparison of M0 versus M0Δ*tprA* strains.** The colour chart illustrates the expression level of each gene based on an average of two experiments (see Materials and Methods). Red indicates the induced genes, and green indicates the genes repressed in the M0 strain. The log2fold-changes in mRNA levels (M0/M0Δ*tprA*) are indicated for each gene by the white number in the boxes. Genes are identified by their unique ID (Gene ID) and are listed in chromosomal order to illustrate the transcriptional units. Genes in bold are located within ICE PAPI-1. The function of genes, known (underlined) or putative, are provided in column 3.

**Figure S7. Identification of promoters directly controlled by TprA.** For each transcriptional unit (TU) detected as differentially expressed by microarray experiment (see Figure 6), EMSA was performed between a 500 bp region just before the TUs supposed to contain TU promoters and TprA purified protein (see Materials and Methods). – : 0 µM TprA, + : 1 µM TprA, B: Bound DNA, F: Free DNA.

**Table S1: Strains used in this study**

| Strains | Relevant characteristics | Origin |
| --- | --- | --- |
| *Escherichia coli* |  |  |
| DH5α | *fhuA2* Δ(*argF-lac*)U169 *phoA* *glnV44* *gyrA96* *recA1 relA1 endA1 thi-1 hsdR17* [Φ80' lacZ(del)M15] | Lab collection |
| SM10 | *thi-1 thr leu tonA lacY supE recA::RP4-2-Tc::Mu (*Km*^R^) supE44* | Lab collection |
| BL21(DE3) | *ompT* *gal* *dcm* *lon* *ΔhsdS* [λ(DE3)] | Lab collection |
| CC118(λpir) | Host strain for pKNG101 replication, Δ(*ara-leu*) *araD* Δl*ac*X74 *galE* *galK phoA20 thi-1 rpsE rpoB* *argE*(Am) *recA1* Rf^R^ (λpir) | Lab collection |
|  |  |  |
| *Pseudomonas aeruginosa* |  |  |
| PA14 | Wild type | Lab collection |
| PAO1*_ppilL2_* | PAO1 strain with *ppilL2-lacZ* fusion inserted at *attB* sites, Tc^R^ | This study |
| PAO1Sm | PAO1 strain carrying streptomycin resistance genes, Sm^R^ | Lab collection |
| PA14*_ppilL2_* | PA14 strain with *ppilL2-lacZ* fusion inserted at *attB* sites, Tc^R^ | This study |
| PA14_GFP_ | PA14 strain tagged with EGFP in a mini Tn7 construct; Sm^R^ | (1) |
| PA14*_ppilL2_*∆*ihfα* | PA14*_ppilL2_* strain carrying the *ihfα* gene with *_T5Stop-K6Stop_* substitution, Tc^R^ | This study |
| PA14*_ppilL2_*∆*ihfβ* | PA14*_ppilL2_* strain carrying the *ihfβ* gene with *_Q32StopM33Stop_* substitution, Tc^R^ | This study |
| PA14*_ppilL2_∆dps* | PA14*_ppilL2_* strain carrying the *dps* gene with *_I7StopG8Stop_* substitution, Tc^R^ | This study |
| PA14*_ppilL2_∆fis* | PA14*_ppilL2_* strain carrying the *fis* gene with *_T8Stop_* substitution, Tc^R^ | This study |
| PA14*_ppilL2_∆hupα* | PA14*_ppilL2_* strain carrying the *hupα* gene with *_A7StopA8Stop_* substitution, Tc^R^ | This study |
| PA14*_ppilL2_∆hupβ* | PA14*_ppilL2_* strain carrying the *hupβ* gene with *_I7StopD8Stop_* substitution, Tc^R^ | This study |
| PA14*_ppilL2_∆mvaT* | PA14*_ppilL2_* strain carrying the *mvaT* gene with *_Y7StopR8Stop_* substitution, Tc^R^ | This study |
| PA14*_ppilL2_∆mvaU* | PA14*_ppilL2_* strain carrying the *mvaU* gene with *_F7StopR8Stop_* substitution, Tc^R^ | This study |
| PA14*_ppilL2∆tprA-BS_-lacZ* | PA14 strain with *ppilL2∆tprA-BS-lacZ* fusion inserted at *attB* sites, Tc^R^ | This study |
| M0 | PA14*_ppilL2_* strain carrying the mariner transposon within the PA14_59030 gene at postion 5,258,903, Gm^R^ | This study |
| M1 | PA14*_ppilL2_* strain carrying the mariner transposon within the *ndpa2* gene at postion 5,259,929, Gm^R^ | This study |
| M2 | PA14*_ppilL2_* strain carrying the mariner transposon within the *tprA* gene at postion 5,260,151, Gm^R^ | This study |
| M3 | PA14*_ppilL2_* strain carrying the mariner transposon within the PA14_59070 gene at postion 5,261,964, Gm^R^ | This study |
| M0*∆ndpA2* | M0 strain carrying the *ndpA2* gene with *_P2Stop_* substitution, Tc^R^ | This study |
| M0*∆tprA* | M0 strain carrying the *tprA* gene with *_A4Stop_* substitution, Tc^R^ | This study |
|  |  |  |

Sm^R^, streptomycin resistance; Gm^R^ gentamicin resistance; Tc^R^ tetracycline resistance.

The position of mariner transposon insertions are given according to the *P. aeruginosa* UCBPP-PA14 genome published under accession number NC_008463.

**Table S2: Plasmids used in this study**

| Plasmids | Relevant characteristics* | Origin |
| --- | --- | --- |
| pRK2013 | *Tra*+ *Mob*+ Km^R^ ; helper plasmid for triparental mating | (2) |
| pMMB67-HE | Broad host range vector, IncQ; *ptac*; *lacZα*, Ap^R^ | Lab collection |
| pKNG101 | Suicide vector in *Pseudomonas* *aeruginosa*, *sacB*^+^, Sm^R^ | Lab collection |
| pBT20 | oriR6K γ, *ptac*, Gm^R^, Ap^R^ | (3) |
| miniCTX-*lacZ* | *lacZ*^+^; self-proficient integration vector with *tet*, V-FRT-attPMCS, *ori*, *int*, and *oriT*, Tc^R^ | (4) |
| miniCTX-*ppilL2-lacZ* | Promoter region of *pilL2* gene inserted into miniCTX-*lacZ*, Tc^R^ | This study |
| miniCTX- *ppilL2∆tprA-BS-lacZ* | Promoter region of *pilL2* gene deleted of the TprA binding site (TprA-BS) inserted into miniCTX-*lacZ*, Tc^R^ | This study |
| pK-M0 | Suicide vector for insertion of mariner transposon within the *PA14_59030* gene by allelic replacement, Sm^R^, Gm^R^ | This study |
| pK-M1 | Suicide vector for insertion of mariner transposon within the *ndpA2* gene by allelic replacement, Sm^R^, Gm^R^ | This study |
| pK-M2 | Suicide vector for insertion of mariner transposon within the *tprA* gene by allelic replacement, Sm^R^, Gm^R^ | This study |
| pK-M3 | Suicide vector for insertion of mariner transposon within the *PA14_59070* gene by allelic replacement, Sm^R^, Gm^R^ | This study |
| pK-*ndpA2_P2Stop_* | Suicide vector for *ndpa2_P2Stop_* substitution by allelic replacement, Sm^R^ | This study |
| pK-*tprA_A4Stop_* | Suicide vector for *tprA_A4Stop_* substitution by allelic replacement, Sm^R^ | This study |
| pK-*ihfα_T5Stop_* | Suicide vector for *ihfα_T5Stop_* substitution by allelic replacement, Sm^R^ | This study |
| pK-*ihfβ_Q32StopM33Stop_* | Suicide vector for *ihfβ_Q32StopM33Stop_* substitution by allelic replacement, Sm^R^ | This study |
| pK-*dps_I7StopG8Stop_* | Suicide vector for *dpsI_7StopG8Stop_* substitution by allelic replacement, Sm^R^ | This study |
| pK-*fis_T8Stop_* | Suicide vector for *fis_T8Stop_* substitution by allelic replacement, Sm^R^ | This study |
| pK-*hupα_A7StopA8Stop_* | Suicide vector for *hupα_A7StopA8Stop_* substitution by allelic replacement Sm^R^ | This study |
| pK-*hupβ_I7StopD8Stop_* | Suicide vector for *hupβ_I7StopD8Stop_* substitution by allelic replacement, Sm^R^ | This study |
| pK-*mvaT_Y7StopR8Stop_* | Suicide vector for *mvaT_Y7StopR8Stop_* substitution by allelic replacement, Sm^R^ | This study |
| pK-*mvaU_F7StopR8Stop_* | Suicide vector for *mvaU_F7StopR8Stop_* substitution by allelic replacement, Sm^R^ | This study |
| pBBR1MCS4 | Broad host range plasmid, Ap^R^ | (5) |
| pBBR-*ndpa2* | pBBR1MCS4 carrying the *ndpa2* gene, Ap^R^ | This study |
| pBBR-*tprA* | pBBR1MCS4 carrying the *tprA* gene, Ap^R^ | This study |
| pBBR-*ndpa2*_*tprA* | pBBR1MCS4 carrying the *ndpa2* and *tprA* genes, Ap^R^ | This study |
| pBBR-*mvaT* | pBBR1MCS4 carrying the *mvaT* gene, Ap^R^ | This study |
| pLIC03 | Vector containing the T7 promoter with pBR322 origin of replication, Km^R^ | (6) |
| pLIC-*tprA* | pLIC03 carrying *tprA* gene, Km^R^ | This study |
| pMMB*pilS2* | *pilS2* gene cloned in pMMB67-HE, Ap^R^ | This study |
|  |  |  |

^*^Sm^R^, streptomycin resistance; Ap^R^, ampicillin resistance; Km^R^, kanamycin resistance; Gm^R^ gentamicin resistance; Tc^R^ tetracycline resistance.

**Table S3. Localisation of transposon insertion during mutagenesis of PA14*_ppilL2_***

| Mutant | Localisation of Tn | *ptac* orientation |
| --- | --- | --- |
|  |  |  |
| Tn2 | *purH* | *purD* |
| Tn4 | *ppilL2-lacZ* fusion | outside *attB* |
| Tn29 | Intergenic region between PA14_58990 and PA14_59000 | PA14_58990 |
| Tn38 | *ppilL2-lacZ* fusion | *lacZ* |
| Tn44 | *ppilL2-lacZ* fusion | *lacZ* |
| Tn48 | *ppilL2-lacZ* fusion | *lacZ* |
| Tn64 | *lolA* | PA14_30320 |
| Tn65 | *ppilL2-lacZ* fusion | *lacZ* |
| Tn67 | *ppilL2-lacZ* fusion | *lacZ* |
| Tn79 | Intergenic region between PA14_35720 and PA14_35730 | PA14_35720 |
| Tn80 | *ppilL2-lacZ* fusion | outside *attB* |
| Tn83 | *purH* | *fis* |
| Tn87 | *ppilL2-lacZ* fusion | *lacZ* |
| Tn89 | *ppilL2-lacZ* fusion | *lacZ* |
| Tn90 | *ppilL2-lacZ* fusion | outside *attB* |
| Tn91 | *ppilL2-lacZ* fusion | *lacZ* |
| Tn93 | PA14_30420 | PA14_30410 |
| Tn94 | *ppilL2-lacZ* fusion | *lacZ* |
| Tn96 | *ppilL2-lacZ* fusion | *lacZ* |
| Tn99 | *ppilL2-lacZ* fusion | *lacZ* |
| Tn104 | *phoU* | *pstB* |
| Tn105 | *phoU* | PA14_70790 |
| Tn107 | *ppilL2-lacZ* fusion | *lacZ* |
| Tn108 | *ppilL2-lacZ* fusion | *lacZ* |
| Tn109 | *ppilL2-lacZ* fusion | *lacZ* |
| Tn113 | *ppilL2-lacZ* fusion | *lacZ* |
| Tn114 | PA14_58990 | PA14_59000 |
| Tn115 | PA14_59030 | PA14_59050 |
| Tn118 | PA14_58990 | PA14_59000 |
| Tn122 | *ppilL2-lacZ* fusion | *lacZ* |
|  |  |  |
|  |  |  |

**Table S4: Function of genes within the PA14_59000-59120 locus**

| Gene ID | Predicted gene functions |
| --- | --- |
| *PA14_58990* | DNA helicase |
| *PA14_59000* | Putative CP11 protein |
| *PA14_59010* | Unknown function |
| *PA14_59020* | Unknown function |
| *PA14_59030* | Unknown function |
| *PA14_59050* | Nucleoid-associated protein, NdpA2 |
| *PA14_59060* | RHH-family transcription factor, TprA |
| *PA14_59070* | Putative ParB family protein |
| *PA14_59090* | DUF2857 domain-containing protein |
| *PA14_59100* | Unknown function |
| *PA14_59110* | Unknown function |
| *PA14_59120* | Unknown function |
|  |  |

**Table S5. Oligonucleotides used for mutagenesis and gene cloning**

| \| Name \| Oligonucleotide (5’→3’) \| Target \| \| --- \| --- \| --- \| \|  \|  \|  \| \| **Transposon mapping** \|  \|  \| \|  \|  \|  \| \| pBT20-1 \| AAGCTGTGGTATGGCTGTGCA \| pBT20 plasmid \| \| pBT20-2 \| AATTGTGAGCGGATAACAATTT \| pBT20 plasmid \| \| ARB1D-Aus \| GGCCAGGCCTGCAGATGATGNNNNNNNNNNGTAT \| pBT20 plasmid \| \| ARB2A-Aus \| GCCAGGCCTGCAGATGATG \| pBT20 plasmid \| \|  \|  \|  \| \| **Mariner insertion** \|  \|  \| \|  \|  \|  \| \| M-1 \| CCACGAATGCGTAACAGGTTGG \| Tn115 genome \| \| M-2 \| GAAGGAAACTAACAGGTTGGC \| Tn115 genome \| \| pK-M0-1 \| CTGCAGGTCGACGGATCCGCGATGCCTGACGTCACAGC \| PA14 genome \| \| pK-M0-2 \| AACCTGTTACGCATTCGTGGTACGCATTCGTGGTCTCCTCG \| PA14 genome \| \| pK-M0-3 \| CCAACCTGTTAGTTTCCTTCGCGGCGCCCGGGAAAGCGTT \| PA14 genome \| \| pK-M0-4 \| TATGGTACCCGGGGATCCGCCGGTGGAGAGATTGGACTCC \| PA14 genome \| \| pK-M1-1 \| CTGCAGGTCGACGGATCCAGTCCAATCTCTCCACCGGCGG \| PA14 genome \| \| pK-M1-2 \| AACCTGTTACGCATTCGTGGTCCTTGTTACGGATGTAGTCGTAGAACG \| PA14 genome \| \| pK-M1-3 \| CCAACCTGTTAGTTTCCTTCCTACGGCCTGTCGCCGGAAATC \| PA14 genome \| \| pK-M1-4 \| TATGGTACCCGGGGATCCCAAGTAGCCGGTCTTTCAGATCTTG \| PA14 genome \| \| pK-M2-1 \| CTGCAGGTCGACGGATCCTCTCGTTCATCAAGGGCAAG \| PA14 genome \| \| pK-M2-2 \| AACCTGTTACGCATTCGTGGCATGGTCATTCTCCAATTTGGGCCTTG \| PA14 genome \| \| pK-M2-3 \| CCAACCTGTTAGTTTCCTTCGCCCGAGAAACCGAAGATAAGTTCGT \| PA14 genome \| \| pK-M2-4 \| TATGGTACCCGGGGATCCATTTGTCCTCTCCAGGGCGTCG \| PA14 genome \| \| pK-M3-1 \| CTGCAGGTCGACGGATCCCTCCGCCACCACCTGTCC \| PA14 genome \| \| pK-M3-2 \| AACCTGTTACGCATTCGTGGGCGAGGCCAGCTGAGAACTGGAATTC \| PA14 genome \| \| pK-M3-3 \| CCAACCTGTTAGTTTCCTTCTCAGTTGCTGCTGGGCCAACC \| PA14 genome \| \| pK-M3-4 \| TATGGTACCCGGGGATCCCCGAAAAAGCTCTGGATCATCGTTGTG \| PA14 genome \| \|  \|  \|  \| \| **Gene inactivation** \|  \|  \| \|  \|  \|  \| \| pK-M0-ndpA2P2Stop-1 \| CTGCAGGTCGACGGATCCGTGGTATGGCTGTGCAGGT \| M0 genome \| \| pK-M0-ndpA2P2Stop-2 \| GGCGTGTTTGATTTACATCAGAAAGACTC \| M0 genome \| \| pK-M0-ndpA2PStop-3 \| GTCTTTCTGATGTAAATCAAACACGCC \| M0 genome \| \| pK-M0- *ndpA2*P2Stop-4 \| TATGGTACCCGGGGATCCCGGAAATAGTCGGAGACCTTCC \| M0 genome \| \| pK-M0-tprAA4Stop-1 \| CTGCAGGTCGACGGATCCTCTCGTTCATCAAGGGCAAGG \| M0 genome \| \| pK-M0-tprAA4Stop-2 \| CGGTTTCGCGCTACATGGTCATTC \| M0 genome \| \| pK-M0-tprAA4Stop-3 \| GACCATGTAGCGCGAAACCGAAG \| M0 genome \| \| pK-M0-tprAA4Stop-4 \| TATGGTACCCGGGGATCCGCCGTTGCGAATGCGG \| M0 genome \| \| pK-ihfα-1 \| CTGCAGGTCGACGGATCCCTTCAGCTTCGTGCCCGGTG \| PA14 genome \| \| pK-ihfα-2 \| AGACGTTCCGCAATTTCAGCTCATTACAGAGCCCCCATACGCTATTTCC \| PA14 genome \| \| pK-ihfα-3 \| AATAGCGTATGGGGGCTCTGTAATGAGCTGAAATTGCGGAACGTCT \| PA14 genome \| \| pK-ihfα-4 \| TATGGTACCCGGGGATCCGGTGAAGCCCTGGTCGTAGAG \| PA14 genome \| \| pK-ihfβ-1 \| CTGCAGGTCGACGGATCCGGGCGACGACATCGAAGGTATCC \| PA14 genome \| \| pK-ihfβ-2 \| CCAGTCGCCAGGGCTTGGGATTATCACTCCAGCATGGTCTTGATTGCCA \| PA14 genome \| \| pK-ihfβ-3 \| CAATCAAGACCATGCTGGAGTGATAATCCCAAGCCCTGGCGACTGG \| PA14 genome \| \| pK-ihfβ-4 \| TATGGTACCCGGGGATCCCCCGCAAGCTGTCAACCTC \| PA14 genome \| \| pK-dps-1 \| CTGCAGGTCGACGGATCCCCAGGCTCTCGTTCAGTTGG \| PA14 genome \| \| pK-dps-2 \| ATGGCTGCGCGATCCTGTTCTTATCATCCGATATTGATTTCCATGGCGTTCTC \| PA14 genome \| \| pK-dps-3 \| CCATGGAAATCAATATCGGATGATAAGAACAGGATCGCGCAGCCAT \| PA14 genome \| \| pK-dps-4 \| TATGGTACCCGGGGATCCCTCAAATCAAGCGGTTGGCG \| PA14 genome \| \| pK-fis-1 \| CTGCAGGTCGACGGATCCCCAGGCTCTTGCGGTACATGG \| PA14 genome \| \| pK-fis-2 \| CACGGGCGTTGTTCCACTCACTAGTTATTCGGTCGTCATCGTTGTCATGC \| PA14 genome \| \| pK-fis-3 \| GCATGACAACGATGACGACCGAATAACTAGTGAGTGGAACAACGCCCG \| PA14 genome \| \| pK-fis-4 \| TATGGTACCCGGGGATCCACTTCCACGGCAGAGATGC \| PA14 genome \| \| pK-hupα-1 \| CTGCAGGTCGACGGATCCTGCTCTATGTGATGCCGCTG \| PA14 genome \| \| pK-hupα-2 \| CTATGCGTAAACCAGAACTATGATAAGCTATCGCCGAAAAGGCCGATC \| PA14 genome \| \| pK-hupα-3 \| GATCGGCCTTTTCGGCGATAGCTTATCATAGTTCTGGTTTACGCATAG \| PA14 genome \| \| pK-hupα-4 \| TATGGTACCCGGGGATCCTTCTTCGGCTTGAGGTACTTCACC \| PA14 genome \| \| pK-hupβ-1 \| CTGCAGGTCGACGGATCCGCCGACGTGGCAATGACCGGGGAGATC \| PA14 genome \| \| pK-hupβ-2 \| GAGTGAACAAGTCGGAACTGTGATAAGCAATTGCCGCATCTGCTGA \| PA14 genome \| \| pK-hupβ-3 \| TCAGCAGATGCGGCAATTGCTTATCACAGTTCCGACTTGTTCACTC \| PA14 genome \| \| pK-hupβ-4 \| TATGGTACCCGGGGATCCCCGCTTCCTTGAGCAGCTTGTC \| PA14 genome \| \| pK-mvaT-1 \| CTGCAGGTCGACGGATCCTGATCCGCCATTCCATGGAGAATTC \| PA14 genome \| \| pK-mvaT-2 \| CTTGATGGCTTCTTCCGTGGCTTATCATTCGTTGATCAGGGACATGTC \| PA14 genome \| \| pK-mvaT-3 \| ATGTCCCTGATCAACGAATGATAAGCCACGGAAGAAGCCATCAAG \| PA14 genome \| \| pK-mvaT-4 \| TATGGTACCCGGGGATCCCAGCGCTATATGGCGAAGAAAG \| PA14 genome \| \| pK-mvaU-1 \| CTGCAGGTCGACGGATCCCAAGGCGATCTTCAAGCCGATC \| PA14 genome \| \| pK-mvaU-2 \| CCTGAAGTTTGCGCTCTGCCTCTTATTACTCGGCAAGTTTGGACATTC \| PA14 genome \| \| pK-mvaU-3 \| GAATGTCCAAACTTGCCGAGTAATAAGAGGCAGAGCGCAAACTTCAGG \| PA14 genome \| \| pK-mvaU-4 \| TATGGTACCCGGGGATCCCGCCATTGTCGCATTCGCGCAG \| PA14 genome \| \|  \|  \|  \| \| **Gene expression** \|  \|  \| \|  \|  \|  \| \| pBBR-ndpA2-1 \| CTGCAGCCCGGGGGATCCAGTCTTTCTGATGCCTATCAAACACGC \| PA14 genome \| \| pBBR-ndpA2-2 \| TCTAGAACTAGTGGATCCCCAAGGGCATACGGACAACGAAC \| PA14 genome \| \| pBBR-tprA-1 \| CTGCAGCCCGGGGGATCCGCGCAAGGCCCAAATTGGAG \| PA14 genome \| \| pBBR-tprA-2 \| TCTAGAACTAGTGGATCCCAGATCTTGCGGGTTGATCTTGG \| PA14 genome \| \|  \|  \|  \| \| **Protein production** \|  \|  \| \|  \|  \|  \| \| pLICc-tprA-1 \| AACCTGTACTTCCAATCAATGATGACCATGGCCCGAGAAACC \| PA14 genome \| \| pLIC-tprA-2 \| TATCCACCTTTACTGTTATTAAGATCTTGCGGGTTGATCTTGG \| PA14 genome \| \| pLICc-mvaT-1 \| ACCTGTACTTCCAATCAATGTCCCTGATCAACGAATATCGC \| PA14 genome \| \| pLICc-mvaT-2 \| ATCCGTATCCACCTTTACTGACTGACTGGTTTAGCCGAGC \| PA14 genome \| \| pLIC-ndpA2-1 \| ACCTGTACTTCCAATCAATGCCTATCAAACACGCCATCG \| PA14 genome \| \| pLIC-ndpA2-2 \| ATCCGTATCCACCTTTACTGCTCGGGCCATGGTCATTCTC \| PA14 genome \| \|  \|  \|  \| \|  \|  \|  \| \| **Chromosomal fusions** \|  \|  \| \|  \|  \|  \| \| MiniCTX-ppilL2-lacZ-1 \| TCCTGCAGCCCGGGGGATCCTGCAGGGGTTTCGGAGAAAAAA \| PA14 genome \| \| MiniCTX-ppilL2-lacZ-2 \| TCGCTAGTTAGTTAGGATCCGTGGTCAACTGCCTGGTCATTG \| PA14 genome \| \| MiniCTX-ppilL2∆tprA-BS-lacZ-1 \| TTAGCCCAATCTGACCTGAGTTCAACAAAG \| MiniCTX-*ppilL2-lacZ* \| \| MiniCTX-ppilL2∆tprA-BS-lacZ-2 \| CTCAGGTCAGATTGGGCTAAATTTTTCTGG \| MiniCTX-*ppilL2-lacZ* \| \|  \|  \|  \| \| **qRT-PCR** \|  \|  \| \|  \|  \|  \| \| RT-07480-1 \| CACTGAAAACCAGGGCAAGC \| PA14_07480 cDNA \| \| RT-07480-2 \| CCTTTTCAACCCTTGTGCCG \| PA14_07480 cDNA \| \| RT-33560-1 \| TCCACCTACGTCGAGACCAT \| PA14_33530 cDNA \| \| RT-33560-2 \| CGCTGTAGTCGCCATAGGAA \| PA14_33530 cDNA \| \| RT-59000-1 \| CCCTCAGCCAAGTGATCTCG \| PA14_59000 cDNA \| \| RT-59000-2 \| GAAGCTCTCGCTCCATCGAA \| PA14_59000 cDNA \| \| RT-59130-1 \| TGTCCGTTACACCGGTCAAG \| PA14_59130 cDNA \| \| RT-59130-2 \| GCGAGTTCGTCGTAGTTGGT \| PA14_59130 cDNA \| \| RT-59180-1 \| AATCTACGTCGCATCGGACC \| PA14_59180 cDNA \| \| RT-59180-2 \| GCAACGCGCTTGTAGTTCTT \| PA14_59180 cDNA \| \| RT-59190-1 \| CAGAGCCGGGATGGGAAAAT \| PA14_59190 cDNA \| \| RT-59190-2 \| TAGTGCTGTGGCTGATTGGG \| PA14_59190 cDNA \| \| RT-pilL2-1 \| GGCCCAGATCATCGACGTAA \| *pilL2* cDNA \| \| RT-pilL2-2 \| TCATCACGTACTGCATGGCG \| *pilL2* cDNA \| \| RT-59380-1 \| CCAAAGCTGAAACGCCAGTC \| PA14_59380 cDNA \| \| RT-59380-2 \| TTTCGCTTTCGGAGTTGGGT \| PA14_59380 cDNA \| \| RT-59390-1 \| GTGGTTATCTGCTCCGGCAT \| PA14_59390 cDNA \| \| RT-59390-2 \| CCGATGTTTTTGGCACCAGG \| PA14_59390 cDNA \| \| RT-59400-1 \| GGCAGACAGCGTGGAATACT \| PA14_59400 cDNA \| \| RT-59400-2 \| TTCTTGGCCTCGACTGCTTC \| PA14_59400 cDNA \| \| RT-59410-1 \| AGGGCAGATACCTGGGCATT \| PA14_59410 cDNA \| \| RT-59410-2 \| TGCTCAGCAGTCAGTTCTGC \| PA14_59410 cDNA \| \| RT-59430-1 \| ACCGTGCTTACTACATCGGC \| PA14_59430 cDNA \| \| RT-59430-2 \| CAAGTTCGCGTGAGGGGTAA \| PA14_59430 cDNA \| \| RT-59440-1 \| CTTGCCCTCGGTGTTGGTAT \| PA14_59440 cDNA \| \| RT-59440-2 \| GACACTCATCCGTCCAAGGG \| PA14_59440 cDNA \| \| RT-59470-1 \| GGTCTATCTCGCCAACCCTG \| PA14_59470 cDNA \| \| RT-59470-2 \| CTGGAGAGAAAGCTCCTGCC \| PA14_59470 cDNA \| \| RT-59480-1 \| ACGAGGAGGTAGAGCGTCTT \| PA14_59480 cDNA \| \| RT-59480-2 \| ACTACTGTGGGGAGCTGGAT \| PA14_59480 cDNA \| \| RT-59520-1 \| TGGTGATGGAAACCTGTCCG \| PA14_59520 cDNA \| \| RT-59520-2 \| AACATCTGGTGCTGGGTGAG \| PA14_59520 cDNA \| \| RT-59640-1 \| GCCTCTGTTTGACGGCAATG \| PA14_59640 cDNA \| \| RT-59640-2 \| CCTGATCGGAGCTGAACGAG \| PA14_59640 cDNA \| \| RT-cupD1-1 \| TACATCTCCGAAGCGACCAC \| *cupD1*  cDNA \| \| RT-cupD1-2 \| ACTCGTAACGCAGGGTGTAG \| *cupD1*  cDNA \| \| RT-59820-1 \| GCTACCTCTACCACAAGGCG \| PA14_59820 cDNA \| \| RT-59820-2 \| GAGCAGGGGATTGATGCTGA \| PA14_59820 cDNA \| \| RT-59860-1 \| ACAGCGCTTCTACTTCGACT \| PA14_59860 cDNA \| \| RT-59860-2 \| TGGGCGTCATGTAGTCCTG \| PA14_59860 cDNA \| \| RT-59980-1 \| GCTGGTCAAGGCACAACAAG \| PA14_59980 cDNA \| \| RT-59980-2 \| TCGACTACTACGGCAGGGAT \| PA14_59980 cDNA \| \| 16S-1 \| CAGCTCGTGTCGTGAGATGT \| 16S cDNA \| \| 16S-2 \| GATCCGGACTACGATCGGTT \| 16S cDNA \| \|  \|  \|  \| \| **EMSA** \|  \|  \| \|  \|  \|  \| \| p07480-1 \| CGGGCAACCTAGGTGAACTGC \| PA14 genome \| \| p07480-2 \| CCGATGGGCTGAGTTCTTTCATGC \| PA14 genome \| \| pPA14_33560-1 \| CGCCAGCAGTCCCATGAAGC \| PA14 genome \| \| pPA14_33560-2 \| GGATGAGCGACGGGAGAACAG \| PA14 genome \| \| pPA14_59000-1 \| AGGAGATGGACTGTCCCGTCG \| PA14 genome \| \| pPA14_59000-2 \| CGGAAAGCGGGCTTCAGTGTTC \| PA14 genome \| \| pPA14_59130-1 \| GGTCGTTCTCGTTCTTTCTGCAC \| PA14 genome \| \| pPA14_59130-2 \| TCTGAAGCCGATGGGTTTCAGC \| PA14 genome \| \| pPA14_59180-1 \| CCTCGTCCTCCGTCGCG \| PA14 genome \| \| pPA14_59180-2 \| TCAATGATTACGATGTGCATCGGC \| PA14 genome \| \| pPA14_59190-1 \| CGTGAAGAACATCATCGACAAGG \| PA14 genome \| \| pPA14_59190-2 \| ATTCTCTTTTCGCATAAACCCTCCTTG \| PA14 genome \| \| ppilL2-1 \| TTGCAGGGGTTTCGGAGAAAAAA \| PA14 genome \| \| ppilL2-2 \| GTGGTCAACTGCCTGGTCATTG \| PA14 genome \| \| pPA14_59380-1 \| CGGATGCGTGCCTACTG \| PA14 genome \| \| pPA14_59380-2 \| CTGGTGGATTGCTTGGACATGG \| PA14 genome \| \| pPA14_59390-1 \| CCAGTCAGGAAGGAGCTCAAGATGG \| PA14 genome \| \| pPA14_59390-2 \| TTTCACTCCTCCTCGGTGAGAGC \| PA14 genome \| \| pPA14_59400-1 \| ATCTTCCTCAGCGAGCACCG \| PA14 genome \| \| pPA14_59400-2 \| GGCTTGGGTGTTGTTGCTCAT \| PA14 genome \| \| pPA14_59410-1 \| GTCCGGCATCGCCAAATTCAT \| PA14 genome \| \| pPA14_59410-2 \| GCATAATCGCCAAACATTTCCCAT \| PA14 genome \| \| pPA14_59430-1 \| GCAAAGGCGAACCTCAAGGTG \| PA14 genome \| \| pPA14_59430-2 \| CCGATGTAGTAAGCACGGTTCTTAT \| PA14 genome \| \| pPA14_59440-1 \| TTTACCCTTTTGCCCATGTGCATCT \| PA14 genome \| \| pPA14_59440-2 \| CGGGTTGAGTTGAGTCATGGG \| PA14 genome \| \| pPA14_59470-1 \| CTTCTGGTGTAACGAGCACGG \| PA14 genome \| \| pPA14_59470-2 \| GCTGGAGTGGTTGTTGAGAGAGG \| PA14 genome \| \| pPA14_59480-1 \| TGCATCCTACCCACCCACGC \| PA14 genome \| \| pPA14_59480-2 \| CCAACTGTCCGGGAACTGTG \| PA14 genome \| \| pPA14_59520-1 \| CGTTCTACTACGGCTGTCCGC \| PA14 genome \| \| pPA14_59520-2 \| GGTAGAGCGGGGTGGGACTG \| PA14 genome \| \| pPA14_59640-1 \| CGATAGTGGCATTGTGGCGG \| PA14 genome \| \| pPA14_59640-2 \| GGGGATGGGCGTTTCATGAG \| PA14 genome \| \| pcupD1-1 \| GTACCTATAGGAGTCCGCACGGTAG \| PA14 genome \| \| pcupD1-2 \| AGGGCAGATCTTTGAGGTTTCATTTCAG \| PA14 genome \| \| pPA14_59820-1 \| CATAGCTTGCCGACTGAGGGAC \| PA14 genome \| \| pPA14_59820-2 \| CCGATCAGCGACCCGAACAT \| PA14 genome \| \| pPA14_59860-1 \| CGTGTCTCTAAACCCGCCAACC \| PA14 genome \| \| pPA14_59860-1 \| CGTTGAGCTGCCGGATCATC \| PA14 genome \| \| R1-1 \| CY5-TTGCAGGGGTTTCGGAGAAAAAACCTGTGACCCAGAACTCTTTTTCCGTATATTTTAAAGCGAAGTCTTCAACAAGCCTTACCGAGAAAGGAAACAGGAT \|  \| \| R1-2 \| ATCCTGTTTCCTTTCTCGGTAAGGCTTGTTGAAGACTTCGCTTTAAAATATACGGAAAAAGAGTTCTGGGTCACAGGTTTTTTCTCCGAAACCCCTGCAA \|  \| \| R2-1 \| CY5-AGTACTGAATCCAGCCACTACCAAGGGGTTAACTGATGCCAGGCTTGGAAATACGGAGCCTTTCCAAGCGACTAAAACAAAGAAGAAAGCTCCCCAGAAA \|  \| \| R2-2 \| TTTCTGGGGAGCTTTCTTCTTTGTTTTAGTCGCTTGGAAAGGCTCCGTATTTCCAAGCCTGGCATCAGTTAACCCCTTGGTAGTGGCTGGATTCAGTACT \|  \| \| R3-1 \| CY5-AATTTAGCCCAATAGTATTTAAAGCTCAATTAGCACCCCGTACTGTCTGACCTGAGTTCAACAAAGCATTCTAAAACGATTAGAACTTCTATTTTATCCA \|  \| \| R3-2 \| TGGATAAAATAGAAGTTCTAATCGTTTTAGAATGCTTTGTTGAACTCAGGTCAGACAGTACGGGGTGCTAATTGAGCTTTAAATACTATTGGGCTAAATT \|  \| \| R3.1-1 \| CY5-AATTTAGCCCAATAGTATTTAAAGCTCAATTAGCAC \|  \| \| R3.1-2 \| GTGCTAATTGAGCTTTAAATACTATTGGGCTAAATT \|  \| \| R3.2-1 \| CY5-AGTATTTAAAGCTCAATTAGCACCCCGTACTGT \|  \| \| R3.2-2 \| ACAGTACGGGGTGCTAATTGAGCTTTAAATACT \|  \| \| R3.3-1 \| CY5-TACTGTCTGACCTGAGTTCAACAAAGCATTCTA \|  \| \| R3.3-2 \| TAGAATGCTTTGTTGAACTCAGGTCAGACAGTA \|  \| \| R3.4-1 \| CY5-ATTCTAAAACGATTAGAACTTCTATTTTATCCA \|  \| \| R3.4-2 \| TGGATAAAATAGAAGTTCTAATCGTTTTAGAAT \|  \| \| R4-1 \| CY5-TTTCTAACAACGATTCCCACCTGGCTCGCTTTAGGCGTAACATGAAAAACCTTCATCGCTCCTAATCCGTTTCCCTTACGCCTGCCTCGCTCACCCTCTG \|  \| \| R4-2 \| CAGAGGGTGAGCGAGGCAGGCGTAAGGGAAACGGATTAGGAGCGATGAAGGTTTTTCATGTTACGCCTAAAGCGAGCCAGGTGGGAATCGTTGTTAGAAA \|  \| \| R5-1 \| CY5-CCTAGTCATCGAGAAATCCCCTCCCCTGTTGCCGAGTATTTCCTGCTGATGCGTGGCGGATGGCCTGCCACTGTGTGCCCCATCATCAAAGGGGGCCTCA \|  \| \| R5-2 \| TGAGGCCCCCTTTGATGATGGGGCACACAGTGGCAGGCCATCCGCCACGCATCAGCAGGAAATACTCGGCAACAGGGGAGGGGATTTCTCGATGACTAGG \|  \| \|  \|  \|  \| |
| --- | --- | --- | --- | --- | --- | --- | --- | --- | --- | --- | --- | --- | --- | --- | --- | --- | --- | --- | --- | --- | --- | --- | --- | --- | --- | --- | --- | --- | --- | --- | --- | --- | --- | --- | --- | --- | --- | --- | --- | --- | --- | --- | --- | --- | --- | --- | --- | --- | --- | --- | --- | --- | --- | --- | --- | --- | --- | --- | --- | --- | --- | --- | --- | --- | --- | --- | --- | --- | --- | --- | --- | --- | --- | --- | --- | --- | --- | --- | --- | --- | --- | --- | --- | --- | --- | --- | --- | --- | --- | --- | --- | --- | --- | --- | --- | --- | --- | --- | --- | --- | --- | --- | --- | --- | --- | --- | --- | --- | --- | --- | --- | --- | --- | --- | --- | --- | --- | --- | --- | --- | --- | --- | --- | --- | --- | --- | --- | --- | --- | --- | --- | --- | --- | --- | --- | --- | --- | --- | --- | --- | --- | --- | --- | --- | --- | --- | --- | --- | --- | --- | --- | --- | --- | --- | --- | --- | --- | --- | --- | --- | --- | --- | --- | --- | --- | --- | --- | --- | --- | --- | --- | --- | --- | --- | --- | --- | --- | --- | --- | --- | --- | --- | --- | --- | --- | --- | --- | --- | --- | --- | --- | --- | --- | --- | --- | --- | --- | --- | --- | --- | --- | --- | --- | --- | --- | --- | --- | --- | --- | --- | --- | --- | --- | --- | --- | --- | --- | --- | --- | --- | --- | --- | --- | --- | --- | --- | --- | --- | --- | --- | --- | --- | --- | --- | --- | --- | --- | --- | --- | --- | --- | --- | --- | --- | --- | --- | --- | --- | --- | --- | --- | --- | --- | --- | --- | --- | --- | --- | --- | --- | --- | --- | --- | --- | --- | --- | --- | --- | --- | --- | --- | --- | --- | --- | --- | --- | --- | --- | --- | --- | --- | --- | --- | --- | --- | --- | --- | --- | --- | --- | --- | --- | --- | --- | --- | --- | --- | --- | --- | --- | --- | --- | --- | --- | --- | --- | --- | --- | --- | --- | --- | --- | --- | --- | --- | --- | --- | --- | --- | --- | --- | --- | --- | --- | --- | --- | --- | --- | --- | --- | --- | --- | --- | --- | --- | --- | --- | --- | --- | --- | --- | --- | --- | --- | --- | --- | --- | --- | --- | --- | --- | --- | --- | --- | --- | --- | --- | --- | --- | --- | --- | --- | --- | --- | --- | --- | --- | --- | --- | --- | --- | --- | --- | --- | --- | --- | --- | --- | --- | --- | --- | --- | --- | --- | --- | --- | --- | --- | --- | --- | --- | --- | --- | --- | --- | --- | --- | --- | --- | --- | --- | --- | --- | --- | --- | --- | --- | --- | --- | --- | --- | --- | --- | --- | --- | --- | --- | --- | --- | --- | --- | --- | --- | --- | --- | --- | --- | --- | --- | --- | --- | --- | --- | --- | --- | --- | --- | --- | --- | --- | --- | --- | --- | --- | --- | --- | --- | --- | --- | --- | --- | --- | --- | --- | --- | --- | --- | --- | --- | --- | --- | --- | --- | --- | --- | --- | --- | --- | --- | --- | --- | --- | --- | --- | --- | --- | --- | --- | --- | --- | --- | --- | --- | --- | --- | --- | --- | --- | --- | --- | --- | --- | --- | --- | --- | --- | --- | --- | --- | --- | --- | --- | --- | --- | --- | --- | --- | --- | --- | --- | --- | --- | --- | --- | --- | --- | --- | --- | --- | --- | --- | --- | --- | --- | --- | --- | --- | --- | --- | --- | --- | --- | --- | --- | --- | --- | --- | --- | --- | --- | --- | --- | --- | --- | --- | --- | --- | --- | --- | --- | --- | --- | --- | --- | --- | --- | --- | --- | --- | --- | --- | --- | --- | --- | --- | --- | --- | --- | --- | --- | --- | --- | --- | --- | --- | --- | --- | --- | --- | --- | --- | --- | --- | --- | --- | --- | --- | --- | --- | --- | --- | --- | --- | --- | --- | --- | --- | --- | --- | --- | --- | --- | --- | --- | --- | --- | --- | --- | --- | --- | --- | --- | --- | --- | --- |

cDNA, complementary DNA; EMSA, electrophoretic mobility shift assay; qRT-PCR, quantitative real-time polymerase chain reaction.

**Supplementary references**

1. Kandalla,P.K., Sarrazin,S., Molawi,K., Berruyer,C., Redelberger,D., Favel,A., Bordi,C., de Bentzmann,S. and Sieweke,M.H. (2016) M-CSF improves protection against bacterial and fungal infections after hematopoietic stem/progenitor cell transplantation. *J. Exp. Med.*, **213**, 2269–2279.

2. Knauf,V.C. and Nester,E.W. (1982) Wide host range cloning vectors: A cosmid clone bank of an Agrobacterium Ti plasmid. *Plasmid*, **8**, 45–54.

3. Kulasekara,H.D., Ventre,I., Kulasekara,B.R., Lazdunski,A., Filloux,A. and Lory,S. (2005) A novel two-component system controls the expression of Pseudomonas aeruginosa fimbrial cup genes. *Mol. Microbiol.*, **55**, 368–380.

4. Hoang,T.T., Kutchma,A.J., Becher,A. and Schweizer,H.P. (2000) Integration-proficient plasmids for Pseudomonas aeruginosa: Site- specific integration and use for engineering of reporter and expression strains. *Plasmid*, **43**, 59–72.

5. Kovach,M.E., Elzer,P.H., Steven Hill,D., Robertson,G.T., Farris,M.A., Roop,R.M. and Peterson,K.M. (1995) Four new derivatives of the broad-host-range cloning vector pBBR1MCS, carrying different antibiotic-resistance cassettes. *Gene*, **166**, 175–176.

6. Stathopulos,J., Cambillau,C., Cascales,E., Roussel,A. and Leone,P. (2015) Crystallization and preliminary X-ray analysis of the C-terminal fragment of PorM, a subunit of the Porphyromonas gingivalis type IX secretion system. *Acta Crystallogr. Sect. FStructural Biol. Commun.*, **71**, 71–74.
