# Supplementary material for: The horizontal transfer of Pseudomonas aeruginosa PA14 ICE PAPI-1 is controlled by a transcriptional triad between TprA, NdpA2 and MvaT
Source: Nucleic Acids Res. 2021 Oct 13;49(19):10956–74. doi: 10.1093/nar/gkab827 (PMC8565334; doi:10.1093/nar/gkab827)
Supplement: gkab827_Supplemental_Files [file gkab827_supplemental_files.zip › Figure S5R2b.pdf]

Differentially expressed genes in M0 strain compared to Tn38 strain of *P. aeruginosa* PA14 (M0 *vs* Tn38)

Gene ID	Log2 fold-change	Function (known or putative)
PA14_05380	1.71	Methyltransferase, PilK
PA14_07470	1.82	tRNA-Met
PA14_07480	6.60	Reverse transcriptase
PA14_10360	1.63	Unknown
PA14_16630	-2.20	Outer membrane protein, OmpA
PA14_16640	-1.81	Lipoprotein
PA14_21260	-2.45	ATP-dependent helicase
PA14_33510	1.98	MbtH family protein
PA14_33540	2.41	ABC transporter permease
PA14_33590	1.88	Thiamine pyrophosphate-binding protein
PA14_33610	1.73	Peptide synthase
PA14_33720	2.69	Protein PvdN
PA14_33730	1.67	Dipeptidase
PA14_33810	3.66	L-ornithine N5-oxygenase
PA14_36650	-1.64	CBS domain-containing protein
PA14_40850	2.06	Histidine phosphatase family protein
PA14_48530	1.61	AMP-binding protein
PA14_50200	1.68	Two-component sensor, FleS
PA14_54840	1.82	tRNA-Gly
**PA14_59030**	6.30	Type II toxin-antitoxin system HicA family toxin
**PA14_59050**	3.00	NdpA-like Anti-histone-like protein, NdpA2
**PA14_59060**	2.34	Transcription factor from RHH-family, TprA
**PA14_59070**	3.72	ParB-like partitionning protein
**PA14_59100**	2.61	Unknown
**PA14_59130**	5.46	TIGR03761 family integrating conjugative element protein
**PA14_59140**	4.73	DUF3158 family protein
**PA14_59150**	3.89	Single stranded DNA binding protein
**PA14_59180**	2.29	Topoisomerase I-family protein
**PA14_59190**	2.07	Unknown
**PA14_59240**	2.43	Type IVb pilus lipoprotein, pilL2
**PA14_59250**	2.50	Type IVb pilus secretin, pilN2
**PA14_59270**	1.94	Type IVb pilus lipoprotein, pilO2
**PA14_59280**	3.62	Type IVb pilus periplasmic protein, pilP2
**PA14_59290**	1.84	Type IVb pilus ATPase, pilQ2
**PA14_59310**	1.95	Type IVb pilus ATPase, pilR2
**PA14_59320**	2.22	Type IVb pilus main prepilin, PilS2
**PA14_59340**	3.77	Type IVb pilus ATPase, PilT2
**PA14_59350**	2.48	Type IVb pilus minor pilin, PilV2
**PA14_59360**	2.00	Type IVb pilus inner membrane protein, PilM2
**PA14_59380**	5.70	Pyrolysyl-tRNA synthetase
**PA14_59390**	3.64	Unknown
**PA14_59400**	7.23	DUF3577 domain-containing protein
**PA14_59410**	6.12	Unknown
**PA14_59430**	5.70	Unknown
**PA14_59440**	3.04	Unknown
**PA14_59470**	4.33	Unknown
**PA14_59480**	6.52	DUF3275 family protein
**PA14_59490**	6.23	Unknown
**PA14_59500**	5.57	Unknown
**PA14_59510**	4.70	Unknown
**PA14_59520**	5.96	Unknown
**PA14_59530**	4.31	Class I SAM-dependent methyltransferase
**PA14_59540**	2.47	DEAD/DEAH box helicase
**PA14_59640**	3.74	Methyl-accepting chemotaxis protein
**PA14_59650**	1.87	TIGR03759 family integrating conjugative element protein
**PA14_59660**	2.40	Lytic transglycosylase
**PA14_59690**	2.42	Type IV conjugative transfer system coupling VirD4-like protein
**PA14_59700**	2.87	TIGR03747 family integrating conjugative element membrane protein
**PA14_59710**	2.82	Fmbriae protein, CupD1
**PA14_59820**	2.87	TIGR03747 family integrating conjugative element membrane protein
**PA14_59860**	1.82	Type III effector Hop protein, RAQPRD family ICE protein
**PA14_59870**	2.73	TIGR03758 family integrating conjugative element protein
**PA14_59880**	3.78	TIGR03745 family integrating conjugative element membrane protein
**PA14_59890**	2.51	TIGR03750 family conjugal transfer protein
**PA14_59900**	1.86	TIGR03746 family integrating conjugative element protein
**PA14_59910**	1.79	TIGR03749 family integrating conjugative element protein
**PA14_59930**	2.95	TIGR03751 family conjugal transfer lipoprotein
**PA14_60000**	2.16	Integrating conjugative element protein
**PA14_60010**	3.39	Unknown
PA14_62400	1.88	Aminotransferase
PA14_62680	-1.64	CsbD family protein
PA14_64480	-1.67	DNA-binding transcriptional activator, OsmE
PA14_65210	1.89	tRNA-Leu
PA14_65220	1.89	tRNA-Leu

Color legend for log2 fold-change :

< -7 0 >7
