# Supplementary material for: The horizontal transfer of Pseudomonas aeruginosa PA14 ICE PAPI-1 is controlled by a transcriptional triad between TprA, NdpA2 and MvaT
Source: Nucleic Acids Res. 2021 Oct 13;49(19):10956–74. doi: 10.1093/nar/gkab827 (PMC8565334; doi:10.1093/nar/gkab827)
Supplement: gkab827_Supplemental_Files [file gkab827_supplemental_files.zip › Figure S6R2b.pdf]

**Differentially expressed genes in M0 strain compared to M0Δ*tprA* strain of *P. aeruginosa* PA14 (M0 *vs* M0Δ*tprA* )**

| Gene ID | log2 fold-change | Function (known or putative) |
|---|---|---|
| PA14_01020 | 1.97 | HsiC1 |
| PA14_01030 | 2.07 | Hcp1 |
| PA14_07470 | 2.59 | tRNA-Met |
| PA14_07480 | 7.16 | Reverse transcriptase |
| PA14_13750 | 2.16 | Nitrite extrusion protein 1 , NarK1 |
| PA14_13770 | 2.11 | Nitrite extrusion protein 2, NarK2 |
| PA14_24650 | -1.82 | ribosome modulation factor, Rmf |
| PA14_33540 | 2.12 | ABC transporter permease |
| PA14_55940 | -2.94 | Type 4b pilus Flp major pilin |
| **PA14_59060** | 4.95 | Transcription factor from RHH-family, TprA |
| **PA14_59070** | 3.24 | ParB-like partitionning protein |
| **PA14_59090** | 1.72 | Unknown |
| **PA14_59100** | 2.22 | Unknown |
| **PA14_59110** | 1.84 | Unknown |
| **PA14_59130** | 3.45 | TIGR03761 family integrating conjugative element protein |
| **PA14_59140** | 3.25 | DUF3158 family protein |
| **PA14_59150** | 2.21 | Single stranded DNA binding protein |
| **PA14_59180** | 2.90 | Topoisomerase I-family protein |
| **PA14_59190** | 2.12 | Unknown |
| **PA14_59210** | 1.65 | DNA helicase |
| **PA14_59220** | 2.29 | pyocin S5 |
| **PA14_59240** | 4.05 | Type IVb pilus lipoprotein, PilL2 |
| **PA14_59250** | 2.50 | Type IVb pilus secretin, PilN2 |
| **PA14_59270** | 2.78 | Type IVb pilus outer membrane protein, PilO2 |
| **PA14_59280** | 3.01 | Type IVb pilus periplasmic protein, PilP2 |
| **PA14_59290** | 3.41 | Type IVb pilus ATPase, PilQ2 |
| **PA14_59310** | 2.95 | Type IVb pilus inner membrane protein, PilR2 |
| **PA14_59320** | 3.45 | Type IVb pilus main prepilin, PilS2 |
| **PA14_59340** | 3.28 | Type IVb pilus ATPase, PilT2 |
| **PA14_59350** | 3.41 | Type IVb pilus minor pilin, PilV2 |
| **PA14_59360** | 2.24 | Type IVb pilus inner membrane protein, PilM2 |
| **PA14_59380** | 6.64 | Pyrolysyl-tRNA synthetase |
| **PA14_59390** | 3.41 | Unknown |
| **PA14_59400** | 5.22 | DUF3577 domain-containing protein |
| **PA14_59410** | 7.29 | Unknown |
| **PA14_59430** | 5.67 | Unknown |
| **PA14_59440** | 3.50 | Unknown |
| **PA14_59470** | 5.02 | Unknown |
| **PA14_59480** | 5.74 | DUF3275 family protein |
| **PA14_59490** | 6.73 | Unknown |
| **PA14_59500** | 7.10 | Unknown |
| **PA14_59510** | 5.33 | Unknown |
| **PA14_59520** | 6.58 | Unknown |
| **PA14_59530** | 4.02 | Class I SAM-dependent methyltransferase |
| **PA14_59640** | 4.04 | Methyl-accepting chemotaxis protein |
| **PA14_59650** | 2.10 | TIGR03759 family integrating conjugative element protein |
| **PA14_59660** | 2.75 | Lytic transglycosylase |
| **PA14_59670** | 2.62 | Integrating conjugative element DUF3577 domain-containing protein |
| **PA14_59680** | 2.00 | dTDP-glucose 4,6-dehydratase |
| **PA14_59690** | 2.57 | Type IV conjugative transfer system coupling VirD4-like protein |
| **PA14_59700** | 3.00 | TIGR03747 family integrating conjugative element membrane protein |
| **PA14_59710** | 3.60 | Fmbriae protein, CupD1 |
| **PA14_59820** | 3.00 | TIGR03747 family integrating conjugative element membrane protein |
| **PA14_59850** | 1.88 | Unknown |
| **PA14_59860** | 2.14 | Type III effector Hop protein, RAQPRD family ICE protein |
| **PA14_59870** | 2.70 | TIGR03758 family integrating conjugative element protein |
| **PA14_59880** | 3.46 | TIGR03745 family integrating conjugative element membrane protein |
| **PA14_59890** | 3.33 | TIGR03750 family conjugal transfer protein |
| **PA14_59900** | 2.90 | TIGR03746 family integrating conjugative element protein |
| **PA14_59910** | 2.62 | TIGR03749 family integrating conjugative element protein |
| **PA14_59930** | 3.07 | TIGR03751 family conjugal transfer lipoprotein |
| **PA14_59940** | 2.48 | Conjugative transfer ATPase VirB4-like protein |
| **PA14_59950** | 1.68 | Unknown |
| **PA14_59960** | 2.75 | DsbA family protein |
| **PA14_59980** | 1.67 | TIGR03757 family integrating conjugative element protein |
| **PA14_59990** | 2.11 | TIGR03756 family integrating conjugative element protein |
| **PA14_60000** | 2.85 | Integrating conjugative element protein |
| **PA14_60010** | 3.52 | Unknown |
| **PA14_60020** | 2.55 | Conjugal transfer protein, TraG |
| PA14_63110 | 1.97 | S-adenosylmethionine decarboxylase |
| PA14_63120 | 2.06 | Fused MFS/spermidine synthase, SpeE |

**Color legend for log2 fold-change :**

< -7 0 >7
