# Supplementary figures and images for: The horizontal transfer of Pseudomonas aeruginosa PA14 ICE PAPI-1 is controlled by a transcriptional triad between TprA, NdpA2 and MvaT
Source: Nucleic Acids Res. 2021 Oct 13;49(19):10956–74. doi: 10.1093/nar/gkab827 (PMC8565334; doi:10.1093/nar/gkab827)

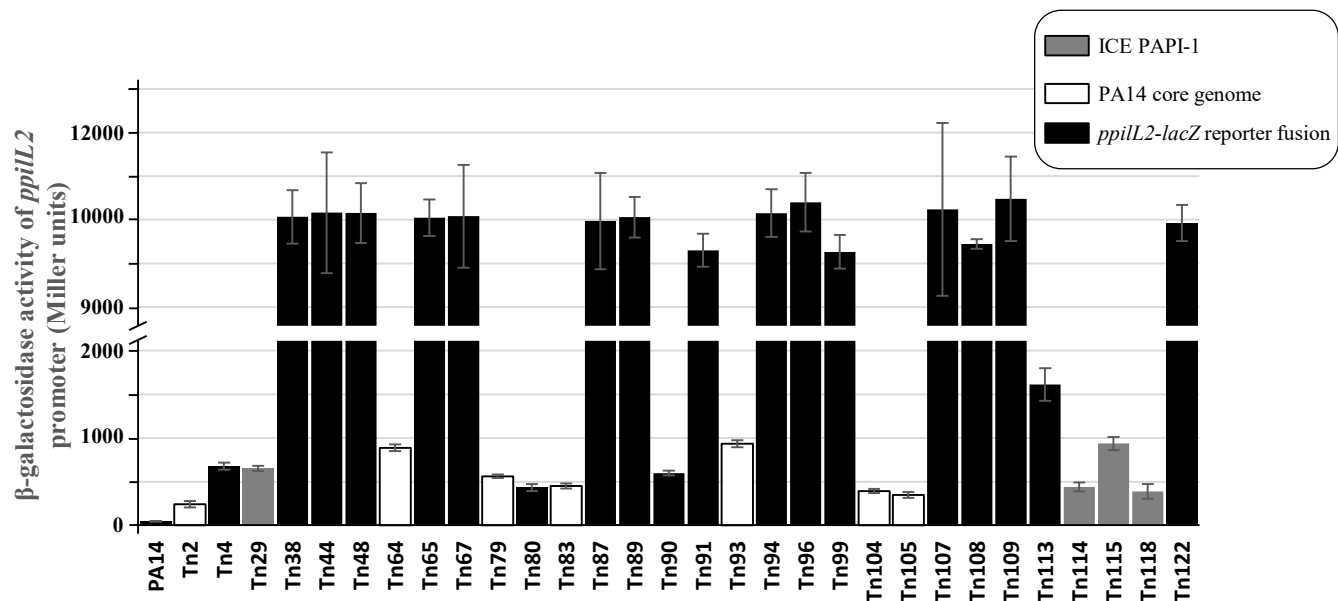

Supplement: gkab827_Supplemental_Files [file gkab827_supplemental_files.zip › Figure S1R2b.pdf]

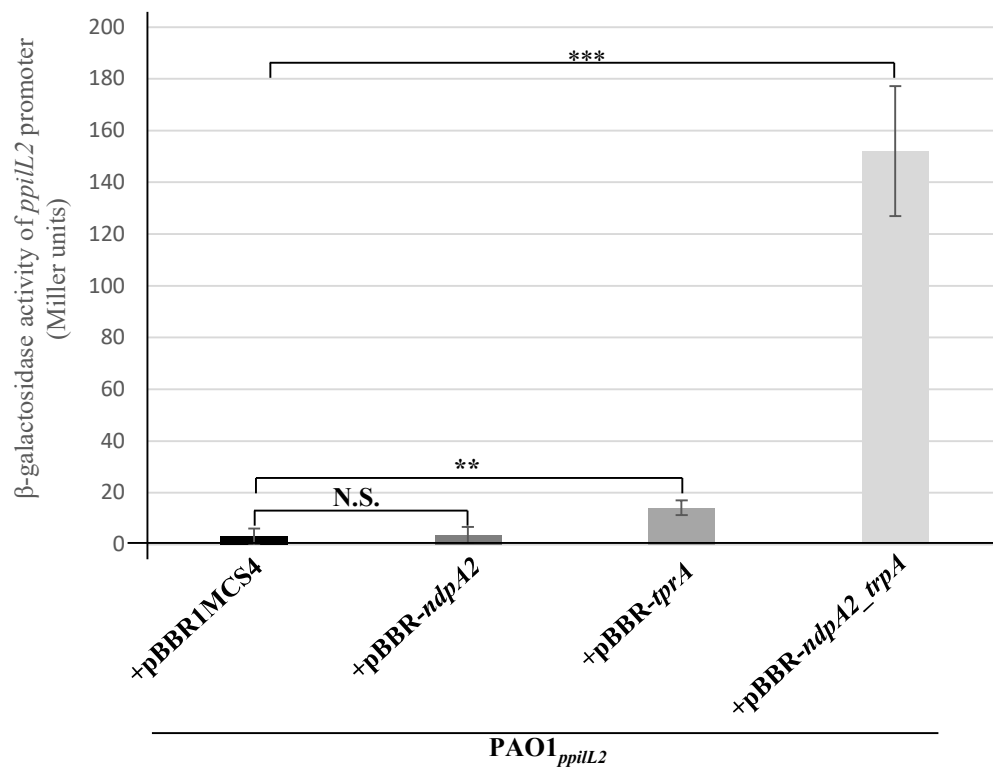

Supplement: gkab827_Supplemental_Files [file gkab827_supplemental_files.zip › Figure S2R2b.pdf]

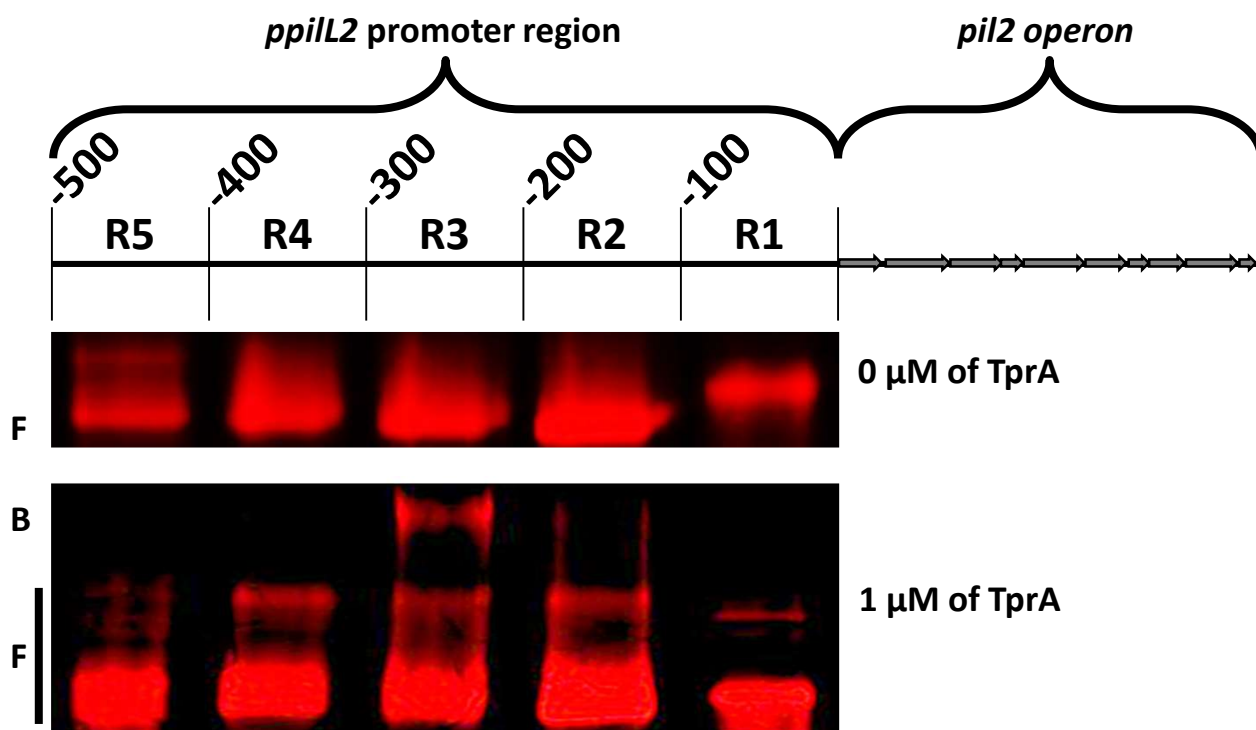

Supplement: gkab827_Supplemental_Files [file gkab827_supplemental_files.zip › Figure S3R2b.pdf]

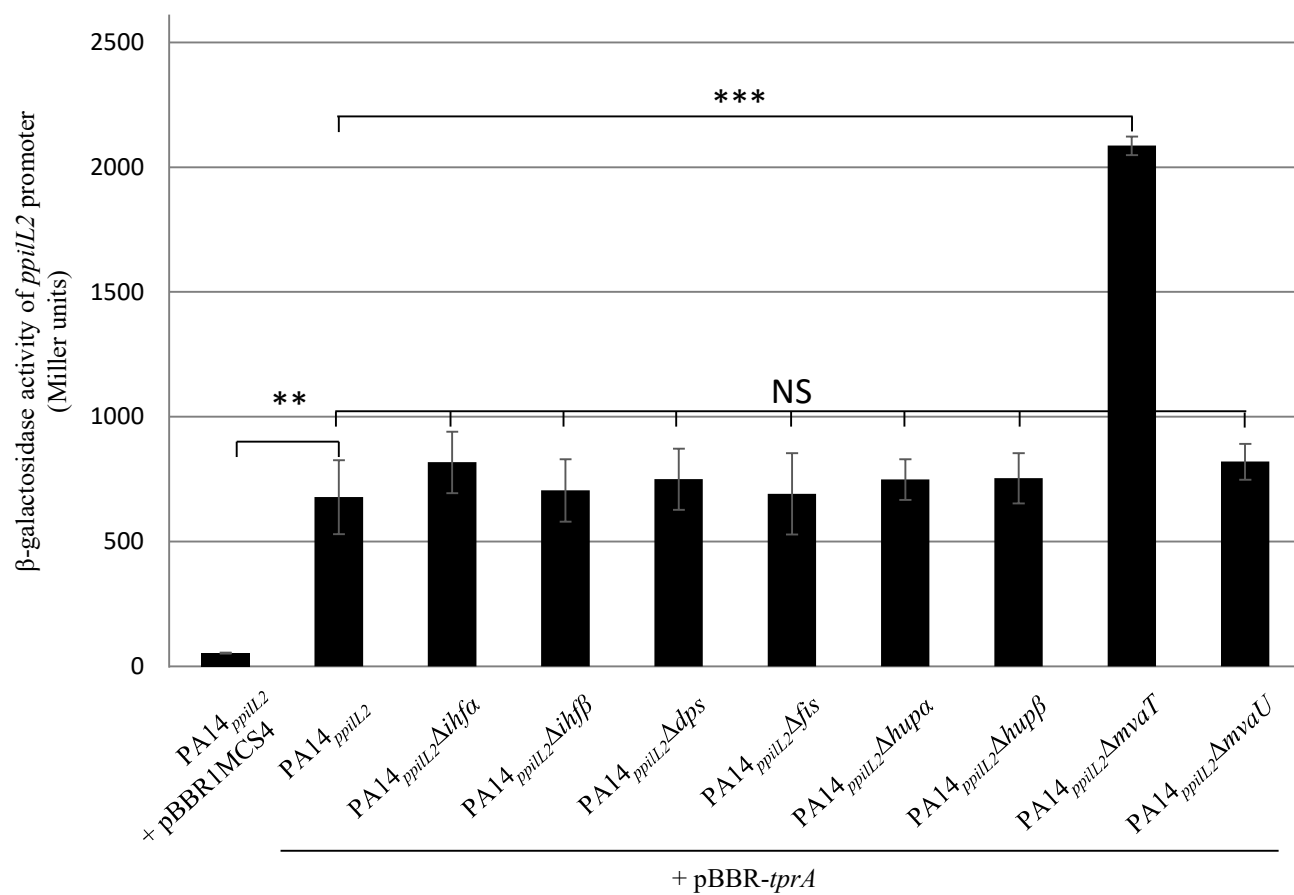

Supplement: gkab827_Supplemental_Files [file gkab827_supplemental_files.zip › Figure S4R2b.pdf]

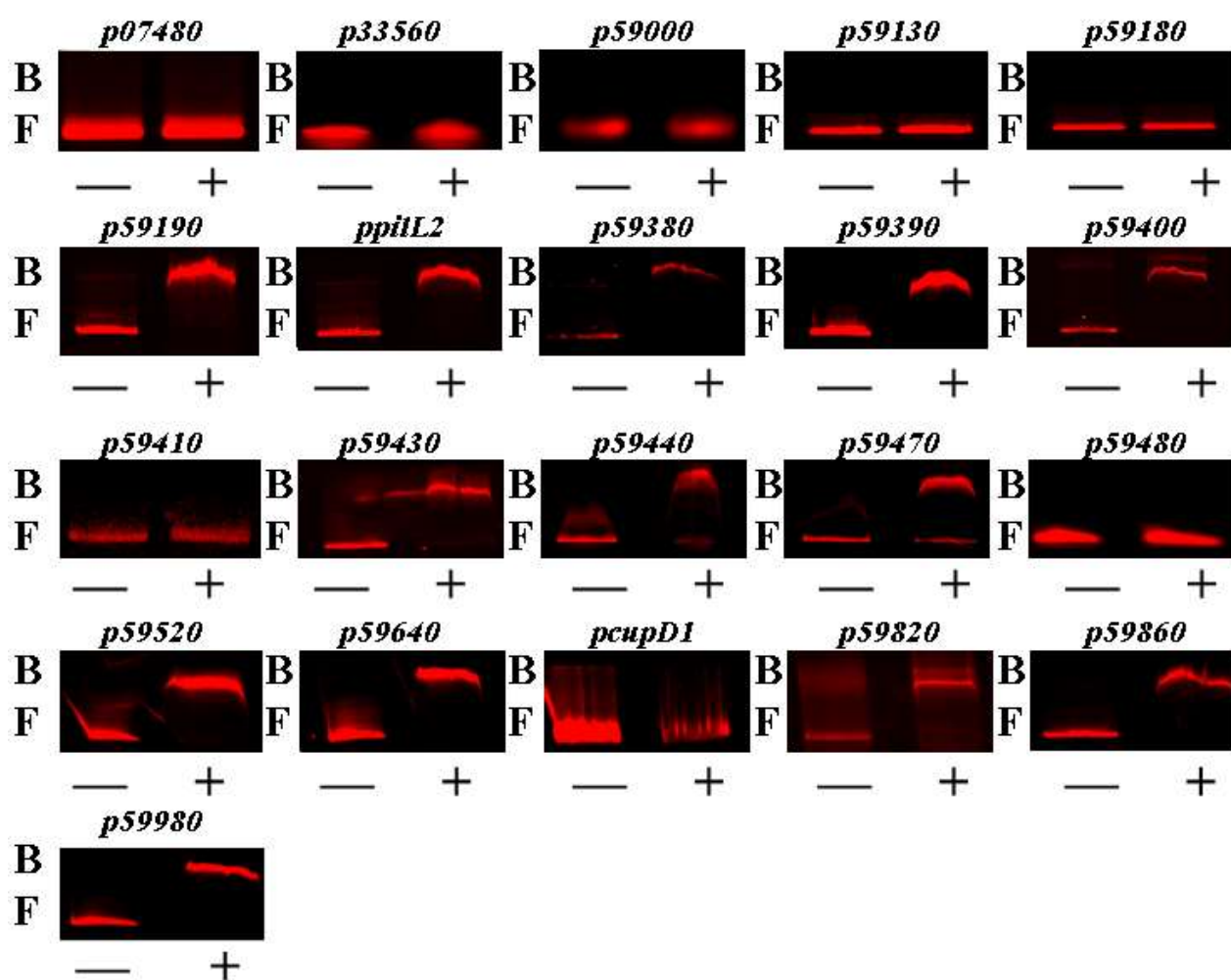

Supplement: gkab827_Supplemental_Files [file gkab827_supplemental_files.zip › Figure S7R2b.pdf]
